# Orbital pacing and secular evolution of the Early Jurassic carbon cycle

**DOI:** 10.1073/pnas.1912094117

**Published:** 2020-02-10

**Authors:** Marisa S. Storm, Stephen P. Hesselbo, Hugh C. Jenkyns, Micha Ruhl, Clemens V. Ullmann, Weimu Xu, Melanie J. Leng, James B. Riding, Olga Gorbanenko

**Affiliations:** ^a^Department of Earth Sciences, Stellenbosch University, 7600 Stellenbosch, South Africa;; ^b^Department of Earth Sciences, University of Oxford, OX1 3AN Oxford, United Kingdom;; ^c^Camborne School of Mines, University of Exeter, TR10 9FE Penryn, United Kingdom;; ^d^Environment and Sustainability Institute, University of Exeter, TR10 9FE Penryn, United Kingdom;; ^e^Department of Geology, Trinity College Dublin, The University of Dublin, Dublin 2, Ireland;; ^f^Department of Botany, Trinity College Dublin, The University of Dublin, Dublin 2, Ireland;; ^g^Environmental Science Centre, British Geological Survey, NG12 5GG Nottingham, United Kingdom;; ^h^School of Biosciences, University of Nottingham, LE12 5RD Loughborough, United Kingdom

**Keywords:** astrochronology, δ^13^CTOC, global carbon cycle, Early Jurassic

## Abstract

Global perturbations to the Early Jurassic environment (∼201 to ∼174 Ma), notably during the Triassic–Jurassic transition and Toarcian Oceanic Anoxic Event, are well studied and largely associated with volcanogenic greenhouse gas emissions released by large igneous provinces. The long-term secular evolution, timing, and pacing of changes in the Early Jurassic carbon cycle that provide context for these events are thus far poorly understood due to a lack of continuous high-resolution δ^13^C data. Here we present a δ^13^C_TOC_ record for the uppermost Rhaetian (Triassic) to Pliensbachian (Lower Jurassic), derived from a calcareous mudstone succession of the exceptionally expanded Llanbedr (Mochras Farm) borehole, Cardigan Bay Basin, Wales, United Kingdom. Combined with existing δ^13^C_TOC_ data from the Toarcian, the compilation covers the entire Lower Jurassic. The dataset reproduces large-amplitude δ^13^C_TOC_ excursions (>3‰) recognized elsewhere, at the Sinemurian–Pliensbachian transition and in the lower Toarcian *serpentinum* zone, as well as several previously identified medium-amplitude (∼0.5 to 2‰) shifts in the Hettangian to Pliensbachian interval. In addition, multiple hitherto undiscovered isotope shifts of comparable amplitude and stratigraphic extent are recorded, demonstrating that those similar features described earlier from stratigraphically more limited sections are nonunique in a long-term context. These shifts are identified as long-eccentricity (∼405-ky) orbital cycles. Orbital tuning of the δ^13^C_TOC_ record provides the basis for an astrochronological duration estimate for the Pliensbachian and Sinemurian, giving implications for the duration of the Hettangian Stage. Overall the chemostratigraphy illustrates particular sensitivity of the marine carbon cycle to long-eccentricity orbital forcing.

## Significance

Cyclic variations in Earth’s orbit drive periodic changes in the ocean–atmosphere system at a time scale of tens to hundreds of thousands of years. The Mochras δ^13^C_TOC_ record illustrates the continued impact of long-eccentricity (405-ky) orbital forcing on the carbon cycle over at least ∼18 My of Early Jurassic time and emphasizes orbital forcing as a driving mechanism behind medium-amplitude δ^13^C fluctuations superimposed on larger-scale trends that are driven by other variables such as tectonically determined paleogeography and eruption of large igneous provinces. The dataset provides a framework for distinguishing between internal Earth processes and solar-system dynamics as the driving mechanism for Early Jurassic δ^13^C fluctuations and provides an astronomical time scale for the Sinemurian Stage.

Prominent carbon-isotope excursions (CIEs) are identified globally in strata from the Triassic–Jurassic boundary (∼201 Ma) and the Toarcian Oceanic Anoxic Event (T-OAE; ∼183 Ma), both of which are expressed in the δ^13^C values derived from various marine and terrestrial organic and inorganic materials (1–3). These isotopic events express changes in the δ^13^C composition of the combined global exogenic carbon pool and are linked to the elevated release of isotopically light volcanic, and/or thermogenic, and/or biogenic carbon into the global ocean–atmosphere system (resulting in negative CIEs, e.g., refs. 4 and 5) and global increase in organic-carbon sequestration in marine and/or terrestrial environments (resulting in positive CIEs, e.g., refs. 6 and 7). Bracketed by these globally recognized distinct large-amplitude δ^13^C events (up to 7‰ in marine and terrestrial δ^13^C_TOC_ records), numerous δ^13^C shifts of somewhat lesser magnitude have been identified in the Hettangian to Pliensbachian interval. Stratigraphically expanded shifts were recorded at the Sinemurian–Pliensbachian boundary (8–14) and the upper Pliensbachian *margaritatus* and *spinatum* zones (10, 15, 16). Furthermore, multiple stratigraphically less extended short-term δ^13^C shifts of ∼0.5 to 2‰ magnitude have been recognized throughout the Hettangian (17–19), in the Sinemurian (17, 20–24), and Pliensbachian (10, 11, 16, 22, 25–28), where they are recorded as individual shifts or series of shifts within stratigraphically limited sections. Some of these short-term δ^13^C excursions have been shown to represent changes in the supraregional to global carbon cycle, marked by synchronous changes in δ^13^C in marine and terrestrial organic and inorganic substrates and recorded on a wide geographic extent (e.g., refs. 10, 16, 23, and 24). However, due to the previous lack of a continuous dataset capturing and contextualizing all isotopic shifts in a single record, there is no holistic understanding of the global nature, causal mechanisms, and the chronology and pacing of these CIEs. Therefore, these δ^13^C shifts have largely been interpreted as stand-alone events, linked to a release of ^12^C from as-yet-undefined sources, reduced organic productivity (leaving more ^12^C in the ocean–atmosphere system) and/or ^13^C-depleted carbon sequestration and orbitally forced environmental change affecting the carbon cycle on the scale of Milankovitch cyclicity (17, 20, 24, 25). Evidence for the latter is so far limited to the Hettangian to early Sinemurian and the early Toarcian, where high-resolution isotope records provide the basis for cyclostratigraphic analysis (17–19, 29–31).

The data illustrated herein provide a continuous and biostratigraphically well-defined δ^13^C_TOC_ record from uppermost Rhaetian (Triassic) to Pliensbachian (Lower Jurassic) strata, with a resolution high enough to examine CIEs of varying magnitudes and temporal extent in their stratigraphic context, thereby enabling a distinction between orbital, tectonic, oceanographic, or volcanic forcing mechanisms of the carbon cycle over this time interval.

## Geological Setting

**Fig. 1.**
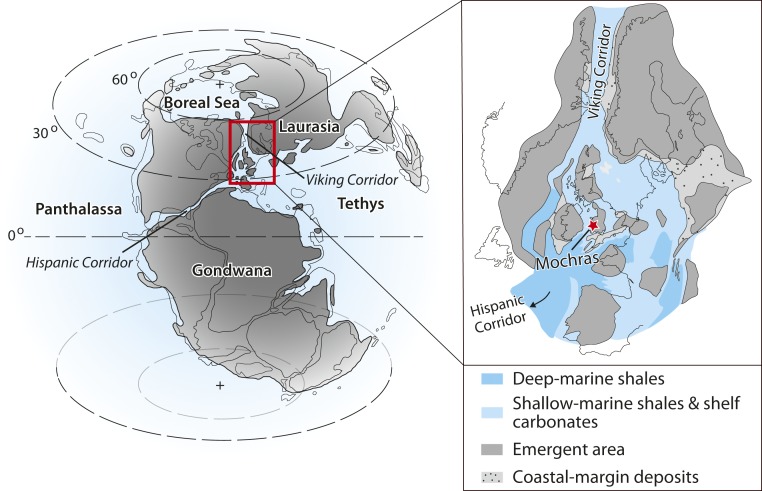
Early Jurassic paleogeography showing the location of the Mochras borehole (red star) within the northern Eurasian Seaway (red rectangle). Reprinted from ref. 39. Copyright (2019) with permission from Elsevier.

The Llanbedr (Mochras Farm) borehole (hereafter referred to as Mochras) cored the Lower Jurassic of the Cardigan Bay Basin (Wales, United Kingdom), an extensional structure related to the breakup of Pangaea (32). In the Early Jurassic, the basin was located at a midpaleolatitude in the Laurasian Seaway on the northwest fringes of the European shelf (Fig. 1 and refs. 33 and 34). The uppermost Pliensbachian and lower Toarcian strata are regarded as having been deposited in an unrestricted, open-marine setting (35).

The recovered sedimentary succession at Mochras comprises 32.05 m of continental Upper Triassic (Rhaetian) deposits (1,938.83 to 1,906.78 m below surface, mbs), ∼1,305 m of Lower Jurassic Hettangian to Toarcian marine strata (1,906.78 to 601.83 mbs), and is unconformably overlain by Paleogene–Neogene sandstones and glaciogenic sediments (601.83 to 0 mbs, ref. 36). Ammonite biostratigraphy of the core was defined to a zonal and even subzonal level, and all ammonite zones of the Lower Jurassic have been identified with the exception of the lowermost Hettangian *tilmanni* zone (37, 38). Due to the lack of the base-Jurassic biostratigraphic marker *Psiloceras spelae*, the Triassic–Jurassic boundary in the Mochras borehole is placed at a lithological change from calcitic dolostone to calcareous mudstone at ∼1,906.78 mbs (36, 38). About 1.7 m of biostratigraphically undefined strata lying between the base Jurassic and the base of the *planorbis* zone are referred to as “pre-*planorbis* beds,” likely equivalent to the basal Jurassic *tilmanni* zone (38).

The relative thinness of the pre-*planorbis* beds suggests a base-Jurassic hiatus at the sharp lithological change at ∼1,906.78 mbs. A calcite-veined interval in the mid-Sinemurian *oxynotum* zone may be marked by a fault which, if present at all, cuts out less than one ammonite subzone (36). A small hiatus may also be present at the level of intraformational conglomerate at 627.38 mbs (36) within the upper Toarcian *pseudoradiosa* zone, and a further unconformity is present at the top of the Lower Jurassic (at 601.85 mbs), where sediments of the uppermost Toarcian *aalensis* zone are overlain by Paleogene strata (7, 36). In all other respects, the Lower Jurassic succession appears to be stratigraphically complete. However, core preservation below ∼1,290 mbs is largely limited to reserve collection samples, each of which aggregate ∼1.4 m intervals of broken core, with consequential reduction of stratigraphic resolution (see *Materials and Methods* and *SI Appendix*).

The Jurassic succession at Mochras is markedly expanded, with relatively uniform lithology compared to coeval strata elsewhere (38, 39). The strata primarily comprise calcareous mudstone, with varying silt and clay content, alternating with strongly bioturbated calcareous siltstone and silty limestone (36). Average Rock-Eval thermal maturation parameter (T_max_ = ∼430 °C) and vitrinite reflectance (R_o_ = 0.38 to 0.63) from previous studies indicate the presence of immature to early mature sedimentary organic matter (7, 22, 40). δ^13^C data from total organic carbon (δ^13^C_TOC_) and carbonates (δ^13^C_carb_) generated in previous studies suggest that the Mochras sedimentary archive records the long-term pattern of global carbon-cycle change (7, 14, 22, 41, 42).

## Results

**Fig. 2.**
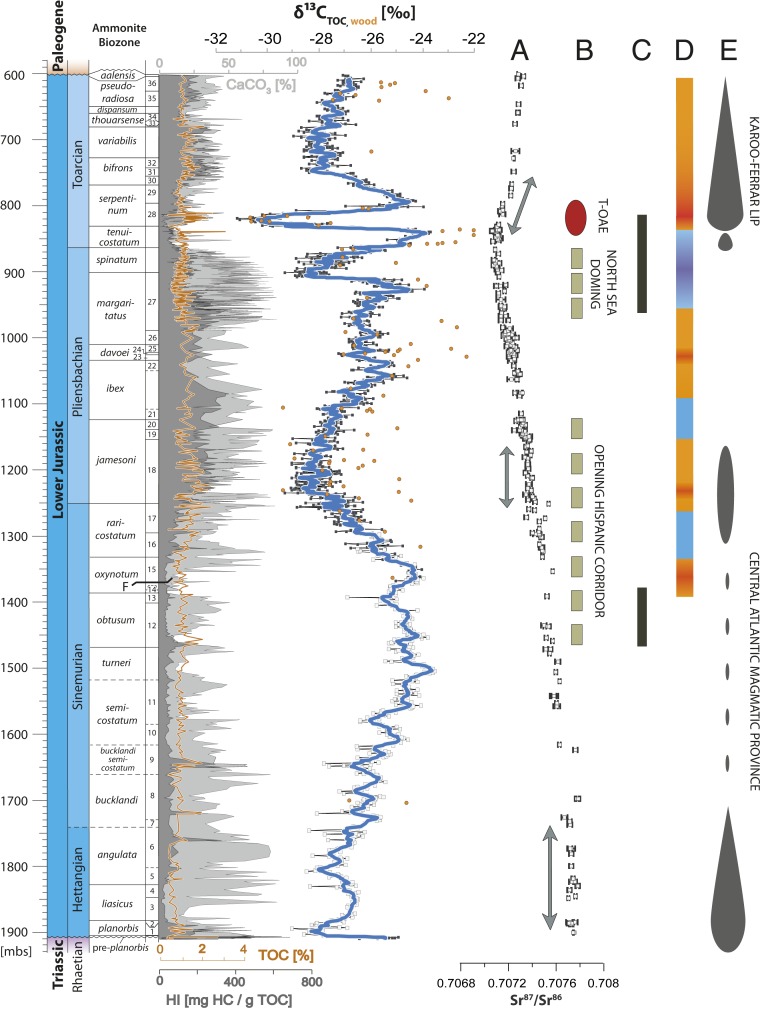
δ^13^C_TOC_, TOC, and CaCO_3_ (calculated from total inorganic carbon), and HI data for the uppermost Rhaetian to Toarcian (ref. 7 and this study) at Mochras. Blue line = seven-point moving average. Black squares = samples taken from core slabs; white squares = samples taken from reserve bags (∼1.4-m intervals) of broken core. Orange circles = δ^13^C_wood_. Depth of the samples from reserve bags refers to midpoint of the sample interval. Ammonite biostratigraphy after refs. 37 and 38. Correlation of the data to paleoenvironmental, oceanic, and magmatic events: (*A*) ^87^Sr/^86^Sr (14, 79). Intervals marked by plateau phases and distinct increases are marked with arrows. (*B*) Paleotectonic events (gray) and T-OAE (red) (6, 51, 80). (*C*) Occurrence of dinoflagellate cysts in the Mochras core (22). (*D*) Paleotemperatures: orange = warming, blue = cooling (9, 49, 51, 81), red = short-lived hyperthermals (21, 24, 48). (*E*) Timing of magmatic events: Central Magmatic Province (based on compilation in ref. 39), Karoo-Ferrar (82). Key for ammonite subzone numbering (question marks indicate uncertainties): 1) *planorbis*, 2) *johnstoni*, 3) *portlock*i, 4) *laqueus*, 5) *extranodosa*, 6) *complanata–depressa?*, 7) *conybeari*, 8) *rotiforme*, 9*) bucklandi*–*lyra*, 10) *scipionianum*, 11) *sauzeanum*, 12) *obtusum*–*stellare*, 13) *denotatus*, 14) *simpsoni*, 15) *oxynotum*, 16) *densinodulum*–*raricostatum*, 17) *macdonnelli*–*aplanatum*, 18) *taylori*–*polymorphus*, 19) *brevispina*, 20) *jamesoni*, 21) *masseanum*?–*valdani*, 22) *luridum*, 23) *maculatum*, 24) *capricornus*, 25) *figulinum*, 26) *stokesi*, 27) *subnodosu*s–*gibbosus*, 28) *exaratum,* 29) *falciferum*, 30) *commune*, 31) *fibulatum*, 32) *crassum*, 33) *fascigerum*, 34) *fallaciosum*, 35) *levesquei*, and 36) *pseudoradiosa.*

The high-resolution δ^13^C_TOC_ and Rock-Eval data from Mochras presented here for the uppermost Rhaetian to Pliensbachian are combined with published data for the Toarcian derived from the same core (ref. 7 and Fig. 2). The compiled δ^13^C_TOC_ record illustrates significant long- and short-term fluctuations in δ^13^C_TOC_ through the Lower Jurassic of the Mochras core. At a longer time scale, the record shows a long-term ∼5‰ positive shift in δ^13^C_TOC_ from the lowermost Hettangian to upper Sinemurian. The Sinemurian–Pliensbachian boundary is characterized by a symmetrically shaped ∼5‰ negative long-term trend and subsequent “recovery” (upper *oxynotum* to upper *ibex* zones, ∼1,360 to ∼1,060 mbs), reaching the lowest values in the lower *jamesoni* zone. The mid-Pliensbachian interval presents a stable plateau in δ^13^C_TOC_, followed by the upper *margaritatus* zone (*subnodosus* and *gibbosus* subzones) where δ^13^C_TOC_ values rise gradually and culminate in an abrupt ∼2‰ positive excursion in the upper *margaritatus* zone (∼930 to ∼926 mbs). The *margaritatus*–*spinatum* zone boundary is marked by a sharp ∼4‰ drop in organic carbon-isotope ratios, followed by a gradual positive shift throughout the *spinatum* zone. The Toarcian record comprises a lower Toarcian overarching positive CIE interrupted by the large negative CIE associated with the T-OAE, as described in previous studies (7, 14, 22, 41). All larger-scale trends in the δ^13^C_TOC_ data are reproduced in δ^13^C_wood_ presented for the upper Sinemurian to Toarcian, although the latter dataset shows a larger degree of variability (Fig. 2).

At a decameter scale, the δ^13^C_TOC_ record is characterized by consecutive alternating positive and negative shifts of ∼0.5 to 2‰ magnitude, superimposed on the observed long-term isotopic trends. These fluctuations in δ^13^C_TOC_, hereafter referred to as medium-amplitude shifts, are particularly well-defined in the Hettangian to uppermost Sinemurian (between 1,906.78 and 1,340 mbs) and the mid-Pliensbachian *ibex* to lower *margaritatus* zones (between 1,120 and 980 mbs). The individual shifts appear larger in magnitude and more stratigraphically extensive in the Hettangian and Sinemurian compared with those in the Pliensbachian. Superimposed on these medium-amplitude shifts, fluctuations in δ^13^C_TOC_ of up to 2‰ on a meter to centimeter scale occur, with larger magnitudes in the upper Sinemurian and Pliensbachian likely being an artifact of differing sample resolution.

The calcium carbonate (CaCO_3_) content of the Mochras strata is highly variable (∼0.6 to 95%; Fig. 2). The long-term shifts in CaCO_3_ appear to negatively correlate with the broad δ^13^C_TOC_ trends, with the exception of the upper Pliensbachian and lower Toarcian successions. On a decameter scale, the CaCO_3_ shows a clear fluctuation in the Hettangian and Sinemurian interval, but the pattern does not correspond to the medium-scale shifts in δ^13^C_TOC._ The relatively higher variability in CaCO_3_ on a meter to decameter scale over the Sinemurian–Pliensbachian transition and the upper Pliensbachian interval is associated with the higher data resolution obtained in these intervals.

The total organic carbon (TOC) content and hydrogen index (HI) values are generally low throughout the Hettangian to Pliensbachian of the Mochras core (Fig. 2). TOC and HI values in the Hettangian and most of the Sinemurian (∼1.5 wt % and ∼80 mg HC/g TOC on average, respectively) notably increase in the upper Sinemurian to lower Pliensbachian (2.6 wt %, up to 380 mg HC/g TOC, respectively) and are moderately elevated through the Pliensbachian (∼1.4 wt % and ∼170 mg HC/g TOC on average, respectively).

The increase in both TOC and HI accompanies the down-going limb of the Sinemurian–Pliensbachian negative CIE, and some stratigraphic intervals with distinctly enhanced TOC and HI values also occur in the Hettangian and Sinemurian, coinciding with minimum values in δ^13^C_TOC_ (for example in the *angulata*, *bucklandi*, and lower *raricostatum* zones). Similarly, the lowermost Jurassic sediments of the pre-*planorbis* beds and *planorbis* zone are also marked by distinctly elevated TOC and HI values (of 3.2 wt % and up to 660 mg HC/g TOC, respectively), coinciding with negative δ^13^C_TOC_ values of −28‰ (Fig. 2). Overall, there is no clear correlation between TOC and δ^13^C_TOC_ (*SI Appendix*, Fig. S5).

Throughout the Toarcian, TOC values fluctuate between 0.5 and 1.5 wt %, with HI values of ∼100 mg HC/g TOC (7). Slightly higher TOC and HI values are recorded in the *bifrons* and *variabilis* zones, and elevated TOC and HI values (up to 2.5 wt % up to 339 mg HC/g TOC, respectively) are associated with the negative CIE interval in the *serpentinum* zone (7).

Predominant components of sedimentary organic matter identified by maceral analysis are liptinites, most of which are represented by liptodetrinite (up to 96.7 vol %; *SI Appendix*, Fig. S7), a product of aerobic and mechanic degradation of liptinitic macerals (43). Markedly smaller but variable amounts of less-degraded liptinite macerals are algal in origin (alginite). Bituminite, also known as amorphous organic matter (AOM), which originates from anaerobic decomposition of algae and faunal plankton under anoxic conditions (44, 45), is primarily present in Pliensbachian samples (up to 24 vol %). Terrestrial organic matter comprising coal clasts, vitrinite, inertinite, sporinite, and cutinite accounts for variable relative amounts (3.3 to 58.8 vol %) of the total organic matter. Notably, the Pliensbachian samples contain a larger relative amount of terrestrially derived organic matter and bituminite compared to the Hettangian and Sinemurian samples. This stratigraphic trend is also reflected in the comparatively high abundance of macrofossil wood in the Pliensbachian and Toarcian part of the core, contrasting with very rare occurrences in the Hettangian and Sinemurian.

## Discussion

### Source and Preservation of Bulk Organic Matter in the Mochras Core.

The formation of liptodetrinite, the predominant organic component in the bulk organic matter assemblage, is associated with physical disintegration of liptinite macerals in the water column and is indicative of extensive water-column circulation and high oxygen availability (45). The precursors of liptodetrinite can be of marine-aquatic origin or derive from terrestrial liptinites, such as sporinite and cutinite (46). The highly degraded shape of liptodetrinite macerals observed in Mochras (*SI Appendix*, Fig. S6) suggests fragile marine organic matter such as algae as primary precursor. The marine origin of the degraded particles is further supported by its fluorescence. The fluorescence of terrestrial organic matter should decrease with greater biodegradation (47) but in the studied samples appears higher compared to intact terrestrial liptinite. The HI values recorded in Mochras are likely highly compromised as a result of aerobic bacterial degradation of initially hydrogen-rich marine organic matter and are therefore not indicative for the primary source of the organic matter.

Comparably larger relative amounts of bituminite (AOM) in the upper Pliensbachian strata indicate more anaerobic bottom-water conditions, resulting in preservation of lipid and hydrogen-rich organic matter. An increase in AOM and foraminifera organic inner wall linings was previously reported from Mochras, concomitant with the increase in TOC in the *raricostatum* to *davoei* zones (22). The elevated TOC and HI values around the Sinemurian–Pliensbachian transition were interpreted to result from both the increase in organic flux to the seafloor and low-oxygen bottom waters (22). Elevated TOC and HI values manifested on a smaller (decameter) scale and stratigraphically coincident with negative CIEs (well-expressed, for example, in the *angulata*, *bucklandi*, and lower *obtusum* zones) may similarly be linked to enhanced preservation of organic matter as a response to redox conditions.

### The Lower Jurassic δ^13^C_TOC_ Record in the Mochras Core.

The large-magnitude CIEs (>3‰) recorded in the Mochras core are the same as isotope events that have been previously recorded elsewhere, such as the early Toarcian negative CIE, punctuating an overarching positive excursion, in the *tenuicostatum*–*serpentinum* zones (7, 14, 22, 41) and the Sinemurian–Pliensbachian boundary negative CIE, both of which have been interpreted as due to increased release of isotopically light carbon into the ocean–atmosphere system (4, 5, 39). Markedly well-expressed in the Mochras δ^13^C_TOC_ record is an upper Pliensbachian (uppermost *margaritatus* zone) negative CIE, revealing a sharp ∼4‰ downward shift following the upper *margaritatus* positive CIE. Both these CIEs have been recognized in multiple European basins (10, 15, 25, 48), as well as in the North American realm (16). The positive *margaritatus* zone CIE has been linked to widespread deposition of isotopically light organic matter during high sea levels and warm climates (6, 25, 49), with no apparent temporal link to large-scale volcanism (16). Subsequent cooling, associated sea-level fall, and restored water-column mixing have been suggested to have released accumulated light carbon through sediment reworking and oxidative and heterotrophic remobilization, causing the negative CIE in the uppermost *margaritatus* zone (8, 10, 26, 50, 51). Similarly, the influx (upwelling/recycling) of ^12^C-rich deep waters associated with a climatic cooling trend has been suggested as an alternative driving mechanism for the Sinemurian–Pliensbachian boundary negative CIE (11).

The clear parallelism between fossil plant matter and bulk organic δ^13^C records demonstrates, however, that the ocean–atmosphere and biosphere carbon reservoirs were simultaneously affected and the shifts in δ^13^C_TOC_ can thus not solely be explained with oceanographic changes such as redox-related preservation or upwelling of ^12^C-enriched deep waters. Despite possible circulation-related redox changes throughout the strata, notably around the Sinemurian–Pliensbachian transition and the upper Pliensbachian (22, 26, 48), the δ^13^C_wood_ record presented here signifies that Mochras appears to record the global δ^13^C evolution.

The most dominant feature illustrated by the Mochras record is, however, the periodic appearance of medium-amplitude (∼0.5 to 2‰) fluctuations on a decameter scale throughout the interval studied, superimposed on the longer-term isotopic shifts discussed above. The medium-amplitude δ^13^C_TOC_ fluctuations appear in a sequence of excursions that are comparable in magnitude and stratigraphic extent. They are expressed throughout the section but appear larger in magnitude in the Hettangian and Sinemurian stratigraphic interval, the deposition of which was associated with more oxygenated bottom waters.

**Fig. 3.**
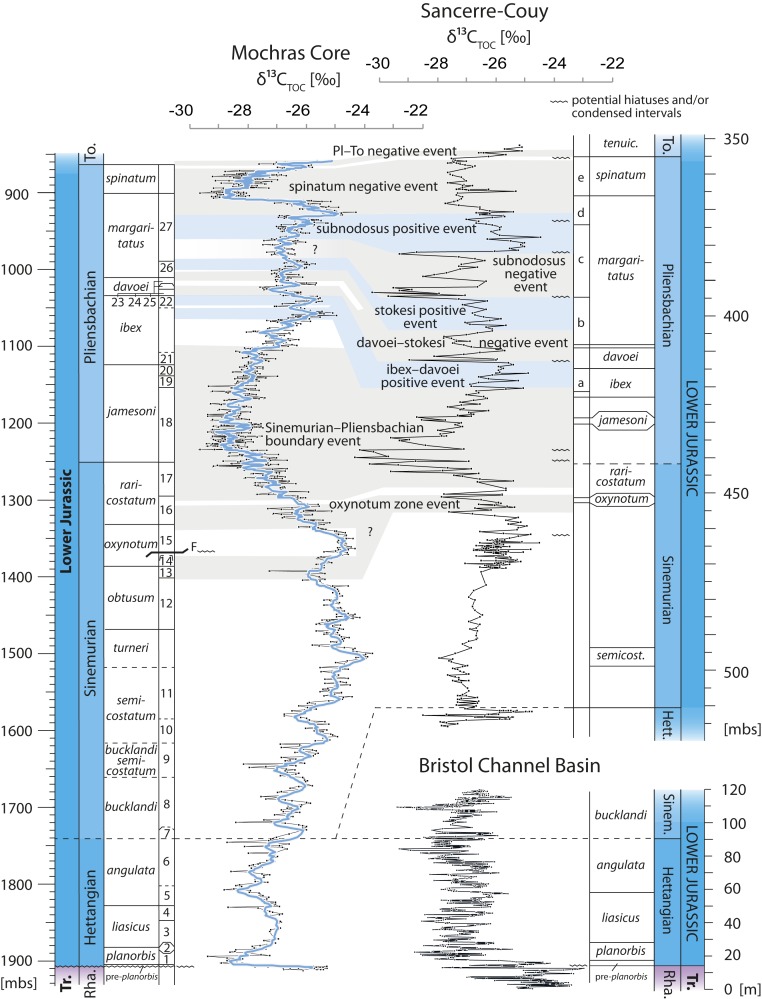
δ^13^C_TOC_ record of the Hettangian to Pliensbachian of the Mochras core, upper Hettangian to Pliensbachian of the Sancerre-Couy core, Paris Basin (15) and the composite Hettangian to lower Sinemurian record from the Bristol Channel Basin (4, 17–19). Key for identified ammonite subzones in Mochras given in Fig. 2. Identified ammonite subzones in the Sancerre-Couy stratigraphy: a. = *valdani*, b. = *stokesi*, c. = *subnodous*, d = *gibbosus*, and e = *solare*. The blue line represents seven-point moving average. Carbon-isotope events identified in Sancerre Couy core are correlated with the Mochras δ^13^C_TOC_ record. Note the differences in stratigraphic resolution and stratigraphic completeness, data resolution, and how some individual medium-amplitude CIEs in the Paris Basin record appear merged into a single event when correlated to Mochras.

A comparison of the Sinemurian and Pliensbachian δ^13^C_TOC_ record of the Sancerre-Couy core (Paris Basin, France; ref. 15), and the Hettangian to lower Sinemurian record from St Audries Bay/East Quantoxhead/Kilve (Bristol Channel Basin, England, United Kingdom; refs. 4 and 17–19), which together represent the hitherto longest high-resolution δ^13^C_TOC_ record of the Lower Jurassic, demonstrates that numerous medium-amplitude δ^13^C_TOC_ shifts recorded in Mochras have previously been merged into what appears as stratigraphically more expanded shifts, or were missed entirely (Fig. 3). The dominant expression of the medium-amplitude CIEs in Mochras likely results from the comparatively high sedimentation rate as well as the high data resolution compared to the Sancerre-Couy record.

Several medium-amplitude CIEs observed in Mochras have previously also been identified in other δ^13^C records covering shorter stratigraphic intervals from geographically widespread sections, and obtained from different carbon substrates, demonstrating that these shifts do not represent a local phenomenon restricted to the Cardigan Bay Basin. In the Sinemurian, for example, a positive CIE in the *turneri* zone is also recorded in δ^13^C_TOC_ on the Dorset coast of the United Kingdom (20) and in western North America (23). A negative CIE in the *obtusum*–*oxynotum* zones is also present in previously published δ^13^C records of bulk organic matter, belemnites, and terrestrially derived palynomorphs from Lincolnshire, United Kingdom, and the δ^13^C_TOC_ records from Robin Hood’s Bay, United Kingdom and Sancerre-Couy, France (15, 21, 24, 42). Although biostratigraphically less well constrained, a similar pattern in δ^13^C_TOC_ has been observed in records from Italy and Morocco (12, 52). Likewise, multiple likely coeval medium-amplitude shifts in δ^13^C within the Pliensbachian *ibex* and *davoei* zones are recorded on a supraregional scale, including the European and African Tethyan margin (9–11, 14, 15, 25, 27, 28, 48, 50, 53). Exceptionally well-expressed are multiple consecutive shifts recorded in δ^13^C_carb_ derived from belemnites from Dorset, United Kingdom, throughout the Pliensbachian *jamesoni*, *ibex*, and lower *davoei* zones (11). Furthermore, multiple shifts in δ^13^C_TOC_ are recorded in the upper Pliensbachian *kunae* and *carlottense* zones of eastern Oregon in the United States, correlative to the mid-*margaritatus* to *spinatum* zones of the northwestern European realm (16).

These CIEs have thus far been discussed as single or episodic events, but as aggregated here in a continuous record and viewed collectively in the overall stratigraphic context they appear as elements in a regular series. Based on the occurrence, in multiple carbon substrates and in various sedimentary basins, it seems apparent that most, if not all, of the medium-amplitude CIEs recorded in the Mochras core reflect changes in the δ^13^C composition of an at least supraregional marine dissolved inorganic carbon pool.

### Pacing of Early Jurassic Carbon-Cycle Fluctuations.

The δ^13^C_TOC_ data presented here point to a common and strongly repetitive, supraregionally to globally acting driving force pacing the observed fluctuations in δ^13^C_TOC_ and concomitant shifts in δ^13^C_carb_. The most plausible driving mechanism acting continuously over an extended time interval is orbital forcing. Compared to shorter Milankovitch periodicities, the long-eccentricity (405-ky) orbital signal can be well expressed in δ^13^C records due to the long residence time of carbon in the ocean–atmosphere system and the associated “memory effect” of carbon in the oceans (54, 55).

Spectral analysis of the Mochras δ^13^C_TOC_ dataset identified dominant spectral peaks, which are changing to slightly higher frequencies up-sequence (*SI Appendix*, Figs. S2 and S3). Average spectral misfit (ASM) testing of these dominant spectral peaks and orbital target frequencies signifies orbital influence as the likely driving mechanism behind the dominant spectral components. The periodicities corresponding to the long-eccentricity (405-ky) cycles visually match the observed medium-amplitude CIEs (*SI Appendix*, Fig. S2) and are in a similar stratigraphic range compared to dominant spectral peaks identified in elemental calcium concentrations and gamma-ray logs identified in the upper Sinemurian to Pliensbachian strata of the same core, which also have been interpreted to represent 405-ky cycles (39, 56).

Thus far, it has not been resolved how eccentricity forcing impacts the carbon cycle and the δ^13^C signature. Orbital eccentricity forcing modulates the precessional amplitude of Earth’s insolation, leading to cyclic changes in seasonal contrasts, with eccentricity maxima marked by high seasonal contrasts and short but intense “monsoon-like” wet intervals followed by prolonged dry periods, whereas eccentricity minima are characterized by more uniform precipitation patterns (57–59). High precipitation and weathering rates during the high-eccentricity wet season is associated with increased continental runoff and fluvial freshwater inputs, as well as increased nutrient and terrestrial organic and inorganic carbon transfer into the oceans, resulting in productivity blooms, a stratified water column, and bottom-water anoxia (58). During the dry season, oxidation of terrestrial organic matter is favored on land, restored water-column mixing oxidizes marine organic matter, and regional carbonate production increases. More stable conditions during eccentricity minima lead to a constant input of freshwater, nutrients, and carbon, resulting in constant productivity rates, persistent watermass stratification, and continuous accumulation of organic-rich deposits in the ocean, as well as increased net production of terrestrial biomass and its storage in stable tropical soils, wetlands, and peats (58, 60, 61). These eccentricity-paced variations in the accumulation and remineralization of marine and terrestrial organic carbon, and the ratio between burial flux of organic carbon and the accumulation rate of inorganic (carbonate) carbon, impact the oceanic carbon pool and are sufficient to drive δ^13^C fluctuations (58, 60–63).

The negative shifts in δ^13^C_TOC_ in Mochras may thus reflect enhanced preservation of isotopically light organic matter in response to orbitally paced redox conditions in the water column and/or bottom waters and sediments. The more distinct expression and larger magnitude of δ^13^C_TOC_ shifts in the Hettangian to upper Sinemurian strata may be linked to the more oxygenated bottom-water conditions that were likely more susceptible to the orbitally paced redox changes.

Increased drawdown of ^12^C-enriched organic matter is, however, generally associated with positive shifts in δ^13^C. Conversely, in Mochras, increased TOC values correspond to negative isotope shifts instead (for example, in the *angulata* and *bucklandi* zones), but overall no correlation between TOC and δ^13^C_TOC_ is apparent. Other Lower Jurassic geochemical records show that medium-amplitude shifts are preceded by, rather than concomitant with, increased TOC intervals (11, 26). This offset may suggest that the accumulation of organic matter took place elsewhere, outside the Cardigan Bay Basin. Furthermore, the long-eccentricity (405-ky) cycles in δ^13^C_TOC_ appear independent of the overall climatic background, as demonstrated by the isotope shifts expressed during the late Pliensbachian, which represents a climatically cold interval (8, 51, 64) and may be less conducive for the development of high seasonal contrasts.

### Early Jurassic Astronomical Time Scale.

**Fig. 4.**
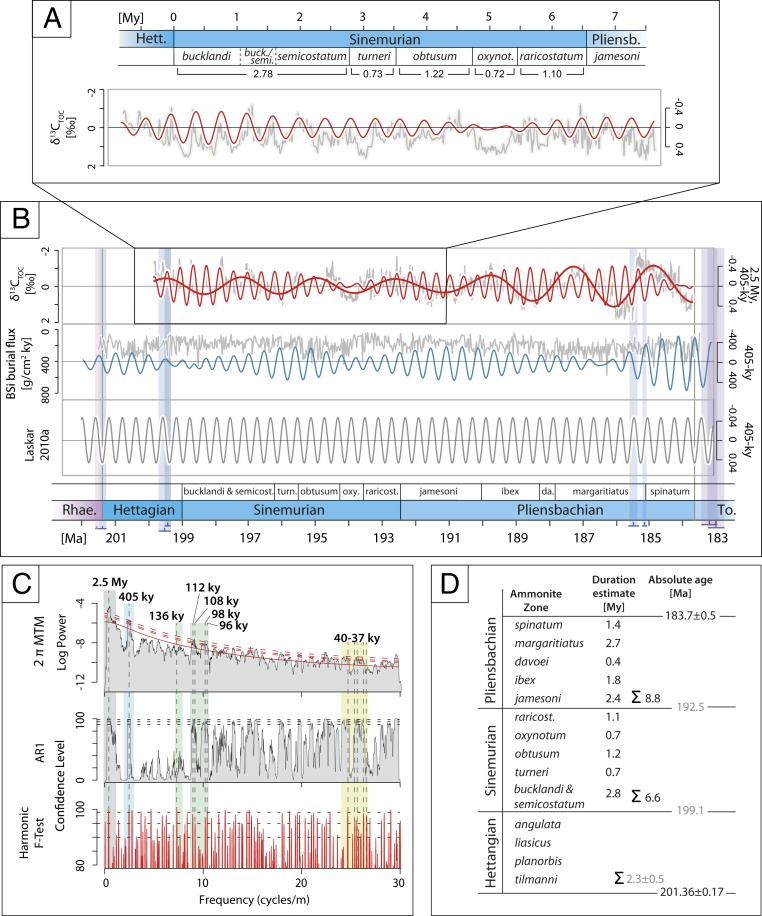
Time-series analysis of the Mochras δ^13^C_TOC_ record. (*A*) The tuned δ^13^C_TOC_ record of the Sinemurian on relative time scale. (*B*) Tuned δ^13^C_TOC_ record of the Sinemurian and Pliensbachian (>3 My frequencies removed), anchored to the Pliensbachian–Toarcian boundary at 183.7 Ma (*Upper*), and 2.5 My and 405-ky band-pass filter, biologic silica burial flux record from Inuyama, Japan (83) and 405-ky band-pass filter (*Middle*) and 405-ky filter of the Laskar astronomical solutions (*Lower* and ref. 84). Absolute radiometric ages from refs. 16, 67, 70–73, 75, 76, 78, and 85. (*C*) Multitaper method (MTM) power spectrum of the tuned δ^13^C_TOC_ Mochras record (frequencies > 3 My removed). Dominant spectral peaks corresponding to 2.5-My amplitude modulations (gray), 405-ky long-eccentricity (blue), and ∼100-ky short-eccentricity (green) and ∼40-ky obliquity (yellow) are marked in figure. (*D*) Summary table of the astronomical duration estimated for the Sinemurian and Pliensbachian Stages and individual ammonite zones. The duration estimate for the Hettangian Stage as well as the stage boundary ages of the Hettangian–Sinemurian and the Sinemurian–Pliensbachian boundaries are inferred and marked in gray.

Due to the stability of the orbital long-eccentricity cycle over the past 215 My (65), orbital tuning of datasets encoding this astronomical metronome can provide reliable time constraints. The 405-ky tuned δ^13^C_TOC_ record from Mochras shows dominant spectral peaks corresponding to amplitude modulation, short eccentricity, and obliquity Milankovitch cycles (Fig. 4). A floating time scale based on the tuned dataset implies a duration of 8.8 My for the Pliensbachian (Fig. 4), which is closely comparable to the cyclostratigraphic duration estimate obtained from elemental calcium concentrations from the same core (8.7 My; ref. 39). The tuned δ^13^C_TOC_ record of the preceding stage provides a direct cyclostratigraphic duration estimate for the Sinemurian and its constituent ammonite zones (Fig. 4). If all cycles were identified correctly, the stage duration of ∼6.6 My defined herein is shorter compared with the previous estimate of 7.6 My, which is based on the assumed linear decrease in ^87^Sr/^86^Sr of belemnite rostra extracted from the Belemnite Marls, Dorset, United Kingdom (66).

Some uncertainty regarding cycle allocation associated with a comparably weak spectral peak in the original and tuned dataset appears in the upper Sinemurian *oxynotum* zone (Fig. 4 and *SI Appendix*, Fig. S2). In this stratigraphic interval, the potential stratigraphic break associated with the possible occurrence of a fault and/or the transition between available sample types and data resolution (transition between samples taken from preserved core slabs and reserve bags) may have led to imprecise cycle allocation. Assuming a relatively uniform sedimentation rate, and considering that the fault, if present, cuts out less than one ammonite subzone, it appears most likely that, if at all, not more than one cycle may be missing in this stratigraphic interval.

Controversial astronomical duration estimates are currently debated for the Hettangian Stage, ranging between 1.7/1.9 My and 4.1 My (17–19, 67, 68). The shorter estimates are derived from multiple astrochronological studies (17–19), with duration estimates from the Hartford Basin, United States, and the biostratigraphically well-constrained St Audries Bay succession, Somerset, United Kingdom, being effectively indistinguishable and supported by correlation to the geomagnetic polarity time scale, as well as radioisotopic ages for the Triassic–Jurassic boundary and earliest Sinemurian from the Pucará Basin, Peru (18, 67, 69–71). The longer estimate of 4.1 My is based on cyclostratigraphic interpretation of spliced magnetic-susceptibility records from multiple UK locations, the individual sections of which suggest durations of 2.9 to 3.2 My (68).

It is possible that the spliced records contain unrecognized stratigraphic overlaps or wrongly identified lithological cycles and thus overestimate the duration of the Hettangian Stage. Equally, however, the individual sections studied for astrochronological constraints may be stratigraphically incomplete and therefore underestimate the time involved in their deposition (68). An additional complication is the possibility that the radioisotopically dated volcanic ashes from the lower Sinemurian in the Pucará Basin in Peru are reworked or correlated inaccurately to the European base-Sinemurian stratotype (68). Temporal constraints on the Hettangian–Sinemurian boundary are thus not entirely resolved.

Although the Hettangian δ^13^C_TOC_ record in Mochras shows consistent medium-amplitude fluctuations similar to the overlying strata, which can be interpreted to represent long-eccentricity forcing, tuning of the Hettangian δ^13^C_TOC_ record in Mochras cannot in itself resolve the duration of the Hettangian Stage as the strata are most likely stratigraphically incomplete at the Triassic–Jurassic boundary.

Biostratigraphically calibrated temporal constraints on the Triassic–Jurassic boundary (201.36 ± 0.17 Ma, recalculated from refs. 70 and 72) are supported by absolute age constraints on the preceding end-Triassic extinction event (73) and the Newark–Hartford astrochronology and geomagnetic polarity time scale (69). The Pliensbachian–Toarcian boundary projected age of 183.7 ± 0.5 Ma (74) is supported by biostratigraphically constrained U-Pb ages from the lower Toarcian (ref. 75, corrected in refs. 76 and 77) and upper Pliensbachian (16, 78). Based on these absolute ages bracketing the studied time interval, the combined Hettangian, Sinemurian, and Pliensbachian Stages cover some 17.7 ± 0.5 My. According to the cyclostratigraphic duration estimates for the Sinemurian and Pliensbachian (∼6.6 and 8.8 My, respectively) given here, the Hettangian Stage is constrained to a duration of ∼2.3 ± 0.5 My. Despite uncertainties, this time scale strongly supports the nonspliced cyclostratigraphic duration estimates for the Hettangian Stage from individual UK sections (17–19, 68).

## Conclusions

The Mochras δ^13^C_TOC_ data from the uppermost Rhaetian to Pliensbachian interval, combined with available data from the Toarcian from the same core, provides a continuous, biostratigraphically well-defined, high-resolution chemostratigraphic record for the Lower Jurassic. Beside large-scale CIEs (>3‰) known from isotopic records elsewhere, CIEs of generally smaller magnitude (0.5 to 2‰) occur throughout the Hettangian to Pliensbachian interval. These medium-amplitude CIEs, including shifts that have previously been recorded in stratigraphically shorter intervals, appear less singular in the context of a continuous record. Spectral and ASM analysis of the data reveals that these medium-amplitude CIEs are paced by long-eccentricity (405-ky) cycles, exemplifying the impact of orbital forcing on the ocean–atmosphere carbon reservoir. Orbital tuning of the isotope record provides a duration estimate of 8.8 My for the Pliensbachian and offers an estimate for the Sinemurian Stage (6.6 My). Combined with published biostratigraphically defined radioisotopic age constraints for the Triassic–Jurassic and Pliensbachian–Toarcian boundaries, the data presented herein suggest a duration for the Hettangian Stage of ∼2.3 ± 0.5 My.

## Materials and Methods

Preserved core slabs, bagged core fragments known as the “reserve collection,” and registered specimens of the Mochras drill core are housed at the British Geological Survey National Geological Repository at Keyworth, United Kingdom. For this study, bulk rock samples between 1,290 and 863.3 mbs were collected from well-preserved core slabs at a 30-cm to 60-cm resolution. Bulk-rock samples below the ∼1,290 mbs level were largely sampled from reserve collections, each of which aggregate ∼1.4 m intervals of broken core. A single sample was taken from each bag and referred to the depth of the midpoint of the sampled interval (reserve bag samples marked as white squares in Fig. 2). Macroscopic fossil plant material was extracted from reserve bags only. The sample resolution, ammonite and foraminiferal biostratigraphy, and a lithological log of the Mochras drill core are shown in *SI Appendix*, Fig. S1.

Detailed information on laboratory procedures for bulk (total) organic carbon-isotope (δ^13^C_TOC_) analyses (1323 samples), fossil plant matter carbon-isotope (δ^13^C_wood_) analysis (95 samples), Rock-Eval pyrolysis (667 samples), organic petrography (14 samples from the Hettangian, Sinemurian, and upper Pliensbachian), and spectral, ASM, and time-series analysis are also given in *SI Appendix*.

### Data Availability Statement.

All data discussed in the paper will be made available in the *SI Appendix* and Dataset S1.

## Supplementary Material

Supplementary File

Supplementary File

## References

[1] Hesselbo S. P., Massive dissociation of gas hydrate during a Jurassic oceanic anoxic event. Nature 406, 392–395 (2000). 10935632 10.1038/35019044

[2] Whiteside J. H., Olsen P. E., Eglinton T., Brookfield M. E., Sambrotto R. N., Compound-specific carbon isotopes from Earth’s largest flood basalt eruptions directly linked to the end-Triassic mass extinction. Proc. Natl. Acad. Sci. U.S.A. 107, 6721–6725 (2010). 20308590 10.1073/pnas.1001706107 PMC2872409

[3] Xu W., Carbon sequestration in an expanded lake system during the Toarcian oceanic anoxic event. Nat. Geosci. 10, 129–134 (2017).

[4] Hesselbo S. P., Robinson S. A., Surlyk F., Piasecki S., Terrestrial and marine extinction at the Triassic-Jurassic boundary synchronized with major carbon-cycle perturbation: A link to initiation of massive volcanism? Geology 30, 251–254 (2002).

[5] McElwain J. C., Wade-Murphy J., Hesselbo S. P., Changes in carbon dioxide during an oceanic anoxic event linked to intrusion into Gondwana coals. Nature 435, 479–482 (2005). 15917805 10.1038/nature03618

[6] Jenkyns H. C., The early Toarcian (Jurassic) anoxic event; Stratigraphic, sedimentary and geochemical evidence. Am. J. Sci. 288, 101–151 (1988).

[7] Xu W., Evolution of the Toarcian (Early Jurassic) carbon-cycle and global climatic controls on local sedimentary processes (Cardigan Bay Basin, UK). Earth Planet. Sci. Lett. 484, 396–411 (2018).

[8] Korte C., Hesselbo S. P., Shallow marine carbon and oxygen isotope and elemental records indicate icehouse-greenhouse cycles during the Early Jurassic. Paleoceanography 26, PA4219 (2011).

[9] Gómez J. J., Comas-Rengifo M. J., Goy A., Palaeoclimatic oscillations in the Pliensbachian (Early Jurassic) of the Asturian Basin (Northern Spain). Clim. Past 12, 1199–1214 (2016).

[10] Mercuzot M., Carbon-isotope events during the Pliensbachian (Lower Jurassic) on the African and European margins of the NW Tethyan Realm. Newsl. Stratigr. 53, 41–69 (2019).

[11] Price G. D., Baker S. J., VanDeVelde J., Clémence M. E., High-resolution carbon cycle and seawater temperature evolution during the Early Jurassic (Sinemurian–Early Pliensbachian). Geochem. Geophys. Geosyst. 17, 3917–3928 (2016).

[12] Franceschi M., Early Pliensbachian (Early Jurassic) C-isotope perturbation and the diffusion of the Lithiotis Fauna: Insights from the western Tethys. Palaeogeogr. Palaeoclimatol. Palaeoecol. 410, 255–263 (2014).

[13] Woodfine R. G., Jenkyns H. C., Sarti M., Baroncini F., Violante C., The response of two Tethyan carbonate platforms to the early Toarcian (Jurassic) oceanic anoxic event: Environmental change and differential subsidence. Sedimentology 55, 1011–1028 (2008).

[14] Jenkyns H. C., Jones C. E., Gröcke D. R., Hesselbo S. P., Parkinson D. N., Chemostratigraphy of the Jurassic System: Applications, limitations and implications for palaeoceanography. J. Geol. Soc. 159, 351–378 (2002).

[15] Peti L., Sinemurian–Pliensbachian calcareous nannofossil biostratigraphy and organic carbon isotope stratigraphy in the Paris Basin: Calibration to the ammonite biozonation of NW Europe. Palaeogeogr. Palaeoclimatol. Palaeoecol. 468, 142–161 (2017).

[16] De Lena L. F., The driving mechanisms of the carbon cycle perturbations in the late Pliensbachian (Early Jurassic). Sci. Rep. 9, 18430 (2019). 31804521 10.1038/s41598-019-54593-1 PMC6895128

[17] Xu W., Ruhl M., Hesselbo S. P., Riding J. B., Jenkyns H. C., Orbital pacing of the Early Jurassic carbon cycle, black-shale formation and seabed methane seepage. Sedimentology 64, 127–149 (2017).

[18] Hüsing S. K., Astronomically-calibrated magnetostratigraphy of the Lower Jurassic marine successions at St. Audrie’s Bay and East Quantoxhead (Hettangian–Sinemurian; Somerset, UK). Palaeogeogr. Palaeoclimatol. Palaeoecol. 403, 43–56 (2014).

[19] Ruhl M., Astronomical constraints on the duration of the early Jurassic Hettangian stage and recovery rates following the end-Triassic mass extinction (St Audrie’s Bay/East Quantoxhead, UK). Earth Planet. Sci. Lett. 295, 262–276 (2010).

[20] Jenkyns H. C., Weedon G. P., Chemostratigraphy (CaCO_3_, TOC, δ^13^C_org_) of Sinemurian (Lower Jurassic) black shales from the Wessex Basin, Dorset and palaeoenvironmental implications. Newsl. Stratigr. 46, 1–21 (2013).

[21] Hesselbo S., Palynological, geochemical, and mineralogical characteristics of the Early Jurassic Liasidium Event in the Cleveland Basin, Yorkshire, UK. Newsl. Stratigr., 10.1127/nos/2019/0536 (2019).

[22] van de Schootbrugge B., Early Jurassic climate change and the radiation of organic-walled phytoplankton in the Tethys Ocean. Paleobiology 31, 73–97 (2005).

[23] Porter S. J., New high resolution geochemistry of Lower Jurassic marine sections in western North America: A global positive carbon isotope excursion in the Sinemurian? Earth Planet. Sci. Lett. 397, 19–31 (2014).

[24] Riding J. B., Leng M. J., Kender S., Hesselbo S. P., Feist-Burkhardt S., Isotopic and palynological evidence for a new Early Jurassic environmental perturbation. Palaeogeogr. Palaeoclimatol. Palaeoecol. 374, 16–27 (2013).

[25] Silva R. L., Duarte L. V., Comas-Rengifo M. J., Mendonça Filho J. G., Azerêdo A. C., Update of the carbon and oxygen isotopic records of the Early–Late Pliensbachian (Early Jurassic,∼187Ma): Insights from the organic-rich hemipelagic series of the Lusitanian Basin (Portugal). Chem. Geol. 283, 177–184 (2011).

[26] Rosales I., Quesada S., Robles S., Geochemical arguments for identifying second-order sea-level changes in hemipelagic carbonate ramp deposits. Terra Nova 18, 233–240 (2006).

[27] Morettini E., Carbon isotope stratigraphy and carbonate production during the Early–Middle Jurassic: Examples from the Umbria–Marche–Sabina Apennines (central Italy). Palaeogeogr. Palaeoclimatol. Palaeoecol. 184, 251–273 (2002).

[28] Quesada S., Robles S., Rosales I., Depositional architecture and transgressive-regressive cycles within Liassic backstepping carbonate ramps in the Basque-Cantabrian basin, northern Spain. J. Geol. Soc. 162, 531–548 (2005).

[29] Sabatino N., Carbon-isotope records of the Early Jurassic (Toarcian) oceanic anoxic event from the Valdorbia (Umbria–Marche Apennines) and Monte Mangart (Julian Alps) sections: Palaeoceanographic and stratigraphic implications. Sedimentology 56, 1307–1328 (2009).

[30] Thibault N., The wider context of the Lower Jurassic Toarcian oceanic anoxic event in Yorkshire coastal outcrops, UK. Proc. Geol. Assoc. 129, 372–391 (2018).

[31] Ait-Itto F.-Z., Martinez M., Price G. D., Ait Addi A., Synchronization of the astronomical time scales in the Early Toarcian: A link between anoxia, carbon-cycle perturbation, mass extinction and volcanism. Earth Planet. Sci. Lett. 493, 1–11 (2018).

[32] Ziegler P. A., Geological Atlas of Western and Central Europe (Shell Internationale Petroleum Maatschappij BV, London, ed. 2, 1990).

[33] Bjerrum C. J., Surlyk F., Callomon J. H., Slingerland R. L., Numerical paleoceanographic study of the Early Jurassic Transcontinental Laurasian Seaway. Paleoceanography 16, 390–404 (2001).

[34] Torsvik T. H., Phanerozoic polar wander, palaeogeography and dynamics. Earth Sci. Rev. 114, 325–368 (2012).

[35] Percival L. M. E., Osmium isotope evidence for two pulses of increased continental weathering linked to Early Jurassic volcanism and climate change. Geology 44, 757–762 (2016).

[36] Woodland A. W., “The Llanbedr (Mochras Farm) Borehole”, A. W. Woodland, Ed. (Rep. No. 71/18, Institute of Geological Sciences, 1971).

[37] Ivimey-Cook H. C., “Stratigraphical palaeontology of the Lower Jurassic of the Llanbedr (Mochras Farm) Borehole” in The Llandbedr (Mochras Farm) Borehole, Woodland A. W., Ed. (Rep. No. 71/18, Institute of Geological Sciences, 1971), pp. 87–92.

[38] Copestake P., Johnson B., Lower Jurassic Foraminifera from the Llanbedr (Mochras Farm) Borehole, North Wales, UK. Monogr. Palaeontogr. Soc. 167, 1–403 (2014).

[39] Ruhl M., Astronomical constraints on the duration of the Early Jurassic Pliensbachian Stage and global climatic fluctuations. Earth Planet. Sci. Lett. 455, 149–165 (2016).

[40] Holford S. P., Green P. F., Turner J. P., Palaeothermal and compaction studies in the Mochras borehole (NW Wales) reveal early Cretaceous and Neogene exhumation and argue against regional Palaeogene uplift in the southern Irish Sea. J. Geol. Soc. 162, 829–840 (2005).

[41] Jenkyns H. C., Clayton C. J., Lower Jurassic epicontinental carbonates and mudstones from England and Wales: Chemostratigraphic signals and the early Toarcian anoxic event. Sedimentology 44, 687–706 (1997).

[42] Katz M. E., Biological overprint of the geological carbon cycle. Mar. Geol. 217, 323–338 (2005).

[43] Taylor G. H., Organic Petrology: A New Handbook Incorporating Some Revised Parts of Stach’s Textbook of Coal Petrology, Glick D. C., Ed. (Gebrüder Borntraeger, Berlin, 1998), p. 704.

[44] Teichmüller M., The genesis of coal from the viewpoint of coal petrology. Int. J. Coal Geol. 12, 1–87 (1989).

[45] Gorbanenko O., Ligouis B., Variations of organo-mineral microfacies of Posidonia Shale from the Lower Saxony Basin and the West Netherlands Basin: Application to paleoenvironmental reconstruction. Int. J. Coal Geol. 152, 78–99 (2015).

[46] Tyson R. V., Sedimentary Organic Matter (Charman & Hall, London, 1995).

[47] Langrock U., Stein R., Origin of marine petroleum source rocks from the Late Jurassic to Early Cretaceous Norwegian Greenland Seaway—evidence for stagnation and upwelling. Mar. Pet. Geol. 21, 157–176 (2004).

[48] Silva R. L., Duarte L. V., Organic matter production and preservation in the Lusitanian Basin (Portugal) and Pliensbachian climatic hot snaps. Global Planet. Change 131, 24–34 (2015).

[49] Suan G., Secular environmental precursors to Early Toarcian (Jurassic) extreme climate changes. Earth Planet. Sci. Lett. 290, 448–458 (2010).

[50] Silva R. L., Duarte L. V., Comas-Rengifo M. J., “Facies and carbon isotope chemostratigraphy of Lower Jurassic carbonate deposits, Lusitanian Basin (Portugal): Implications and limitations to the application in sequence stratigraphic studies” in Chemostratigraphy, Ramkumar M., Ed. (Elsevier, 2015), pp. 341–371.

[51] Korte C., Jurassic climate mode governed by ocean gateway. Nat. Commun. 6, 10015 (2015). 26658694 10.1038/ncomms10015 PMC4682040

[52] Danisch J., Kabiri L., Nutz A., Bodin S., Chemostratigraphy of late Sinemurian–early Pliensbachian shallow-to deep-water deposits of the Central High Atlas Basin: Paleoenvironmental implications. J. Afr. Earth Sci. 153, 239–249 (2019).

[53] Rosales I., Quesada S., Robles S., Primary and diagenetic isotopic signals in fossils and hemipelagic carbonates: The Lower Jurassic of northern Spain. Sedimentology 48, 1149–1169 (2001).

[54] Cramer B. S., Wright J. D., Kent D. V., Aubry M. P., Orbital climate forcing of δ^13^C excursions in the late Paleocene–early Eocene (chrons C24n–C25n). Paleoceanogr. Paleoclimatol. 18, 1097 (2003).

[55] Pälike H., The heartbeat of the Oligocene climate system. Science 314, 1894–1898 (2006). 17185595 10.1126/science.1133822

[56] Hesselbo S. P., Mochras borehole revisited: A new global standard for Early Jurassic earth history. Sci. Drill. 16, 81–91 (2013).

[57] Paillard D., Climate and the orbital parameters of the Earth. C. R. Geosci. 342, 273–285 (2010).

[58] Martinez M., Dera G., Orbital pacing of carbon fluxes by a ∼9-My eccentricity cycle during the Mesozoic. Proc. Natl. Acad. Sci. U.S.A. 112, 12604–12609 (2015). 26417080 10.1073/pnas.1419946112 PMC4611626

[59] Valdes P. J., Glover R. W., Modelling the climate response to orbital forcing. Philos. Trans. Royal Soc. Lond. Ser. A 357, 1873–1890 (1999).

[60] Ma W., Tian J., Li Q., Wang P., Simulation of long eccentricity (400-kyr) cycle in ocean carbon reservoir during Miocene Climate Optimum: Weathering and nutrient response to orbital change. Geophys. Res. Lett. 38, L10701 (2011).

[61] Zachos J. C., McCarren H., Murphy B., Röhl U., Westerhold T., Tempo and scale of late Paleocene and early Eocene carbon isotope cycles: Implications for the origin of hyperthermals. Earth Planet. Sci. Lett. 299, 242–249 (2010).

[62] Silva R. L., Duarte L. V., Wach G. D., Morrison N., Campbell T., Oceanic organic carbon as a possible first-order control on the carbon cycle during the Bathonian–Callovian. Global Planet. Change 184, 103058 (2020).

[63] Locklair R., Sageman B., Lerman A., Marine carbon burial flux and the carbon isotope record of Late Cretaceous (Coniacian–Santonian) Oceanic Anoxic Event III. Sediment. Geol. 235, 38–49 (2011).

[64] Alberti M., Fürsich F. T., Andersen N., First steps in reconstructing Early Jurassic sea water temperatures in the Andean Basin of northern Chile based on stable isotope analyses of oyster and brachiopod shells. J. Palaeogeogr. 8, 33 (2019).

[65] Kent D. V., Empirical evidence for stability of the 405-kiloyear Jupiter-Venus eccentricity cycle over hundreds of millions of years. Proc. Natl. Acad. Sci. U.S.A. 115, 6153–6158 (2018). 29735684 10.1073/pnas.1800891115 PMC6004457

[66] Weedon G. P., Jenkyns H. C., Coe A. L., Hesselbo S. P., Astronomical calibration of the Jurassic time-scale from cyclostratigraphy in British mudrock formations. Philos. Trans. Royal Soc. Lond. Ser. A 357, 1787–1813 (1999).

[67] Schaltegger U., Guex J., Bartolini A., Schoene B., Ovtcharova M., Precise U–Pb age constraints for end-Triassic mass extinction, its correlation to volcanism and Hettangian post-extinction recovery. Earth Planet. Sci. Lett. 267, 266–275 (2008).

[68] Weedon G. P., Page K. N., Jenkyns H. C., Cyclostratigraphy, stratigraphic gaps and the duration of the Hettangian Stage (Jurassic): Insights from the Blue Lias Formation of southern Britain. Geol. Mag. 156, 1469–1509 (2019).

[69] Kent D. V., Olsen P. E., Muttoni G., Astrochronostratigraphic polarity time scale (APTS) for the Late Triassic and Early Jurassic from continental sediments and correlation with standard marine stages. Earth Sci. Rev. 166, 153–180 (2017).

[70] Wotzlaw J. F., Towards accurate numerical calibration of the Late Triassic: High-precision U-Pb geochronology constraints on the duration of the Rhaetian. Geology 42, 571–574 (2014).

[71] Guex J., Geochronological constraints on post-extinction recovery of the ammonoids and carbon cycle perturbations during the Early Jurassic. Palaeogeogr. Palaeoclimatol. Palaeoecol. 346–347, 1–11 (2012).

[72] Schoene B., Guex J., Bartolini A., Schaltegger U., Blackburn T. J., Correlating the end-Triassic mass extinction and flood basalt volcanism at the 100 ka level. Geology 38, 387–390 (2010).

[73] Blackburn T. J., Zircon U-Pb geochronology links the end-Triassic extinction with the Central Atlantic Magmatic Province. Science 340, 941–945 (2013). 23519213 10.1126/science.1234204

[74] Ogg J. G., Ogg G. M., Gradstein F. M., “Jurassic” in A Concise Geologic Time Scale, Ogg J. G., Ogg G. M., Gradstein F. M., Eds. (Elsevier, 2016), chap. 12, pp. 167–186.

[75] Mazzini A., Svensen H., Leanza H. A., Corfu F., Planke S., Early Jurassic shale chemostratigraphy and U–Pb ages from the Neuquén Basin (Argentina): Implications for the Toarcian Oceanic Anoxic event. Earth Planet. Sci. Lett. 297, 633–645 (2010).

[76] Corfu F., Svensen H., Mazzini A., Comment to paper: Evaluating the temporal link between the Karoo LIP and climatic–biologic events of the Toarcian Stage with high-precision U–Pb geochronology by Bryan Sell, Maria Ovtcharova, Jean Guex, Annachiara Bartolini, Fred Jourdan, Jorge E. Spangenberg, Jean-Claude Vicente, Urs Schaltegger in Earth and Planetary Science Letters 408 (2014) 48–56. Earth Planet. Sci. Lett. 434, 349–352 (2016).

[77] Al-Suwaidi A. H., The Toarcian Oceanic Anoxic Event (Early Jurassic) in the Neuquén Basin, Argentina: A reassessment of age and carbon isotope stratigraphy. J. Geol. 124, 171–193 (2016).

[78] Them T. R., Evidence for rapid weathering response to climatic warming during the Toarcian Oceanic Anoxic Event. Sci. Rep. 7, 5003 (2017). 28694487 10.1038/s41598-017-05307-y PMC5504049

[79] Jones C. E., Jenkyns H. C., Hesselbo S. P., Strontium isotopes in Early Jurassic seawater. Geochim. Cosmochim. Acta 58, 1285–1301 (1994).

[80] Aberhan M., Opening of the Hispanic Corridor and Early Jurassic bivalve biodiversity. Geol. Soc. Lond. Spec. Publ. 194, 127–139 (2002).

[81] Bougeault C., Climatic and palaeoceanographic changes during the Pliensbachian (Early Jurassic) inferred from clay mineralogy and stable isotope (C-O) geochemistry (NW Europe). Global Planet. Change 149, 139–152 (2017).

[82] Duncan R. A., Hooper P. R., Rehacek J., Marsh J. S., Duncan A. R., The timing and duration of the Karoo igneous event, southern Gondwana. J. Geophys. Res. Solid Earth 102, 18127–18138 (1997).

[83] Ikeda M., Tada R., Ozaki K., Astronomical pacing of the global silica cycle recorded in Mesozoic bedded cherts. Nat. Commun. 8, 15532 (2017). 28589958 10.1038/ncomms15532 PMC5467233

[84] Laskar J., Fienga A., Gastineau M., Manche H., La2010: A new orbital solution for the long-term motion of the Earth. Astron. Astrophys. 532, A89 (2011).

[85] Sell B., Evaluating the temporal link between the Karoo LIP and climatic–biologic events of the Toarcian Stage with high-precision U–Pb geochronology. Earth Planet. Sci. Lett. 408, 48–56 (2014).

