# Supplementary material for: Orbital pacing and secular evolution of the Early Jurassic carbon cycle
Source: Proc Natl Acad Sci U S A. 2020 Feb 10;117(8):3974–82. doi: 10.1073/pnas.1912094117 (PMC7049106; doi:10.1073/pnas.1912094117)
Supplement: Supplementary File [file pnas.1912094117.sapp.pdf]

SUPPLEMENTARY INFORMATION

Orbital pacing and secular evolution of the Early Jurassic carbon cycle

Marisa S. Storm[1,2], Stephen P. Hesselbo[3], Hugh C. Jenkyns[2], Micha Ruhl[2,4], Clemens V. Ullmann[3], Weimu Xu[2,5], Melanie J. Leng[6,7], James B. Riding[6], Olga Gorbanenko[2]

[1] *Department of Earth Sciences, Stellenbosch University, Stellenbosch 7600, South Africa,* phone: +27 21 808 4627, Email: *marisastorm@sun.ac.za*

[2] *Department of Earth Sciences, University of Oxford, Oxford OX1 3AN, UK*

[3] *Camborne School of Mines and Environment and Sustainability Institute, University of Exeter, Penryn TR10 9FE, UK*

[4] *Department of Geology, Trinity College Dublin, The University of Dublin, Dublin 2, Ireland*

[5] *Department of Botany, Trinity College Dublin, The University of Dublin, Dublin 2, Ireland*

[6] *British Geological Survey, Keyworth, Nottingham NG12 5GG, UK*

[7] *School of Biosciences, University of Nottingham, Loughborough, LE12 5RD, UK*

1. MATERIALS

1.1 MATERIALS

The Llanbedr (Mochras Farm) borehole is located at 52°48’32’’N, 4°08’44’’W, on a coastal dune field west of the village of Llanbedr in Gwynedd, west Wales, UK (1). Preserved core-slabs, reserve collections, bagged fragments (reserve bags), registered specimens and museum specimens are housed at the British Geological Survey (BGS) National Geological Repository (NGR) in Keyworth, Nottingham, UK. For this study, samples between ~1300 and 863.3 m below surface (mbs) were collected from well-preserved core slabs. Samples below ~1300 mbs were sampled from the reserve collection samples or from core slabs where available. The reserve collection samples each aggregate broken core fragments from ~1.4 m intervals. A single sample was taken from each bag, referring to the depth of the mid-point of the sampled interval (samples marked as red squares in Fig. 2 of the main text). Macroscopic fossil plant material was extracted from reserve collection samples. Core preservation, sample resolution of individual sampling intervals, ammonite and foraminiferal biostratigraphy and a lithological log of the Mochras drill-core are shown in Figure S1.

2. METHODS

2.1 ROCK-EVAL PYROLYSIS

Rock-Eval analysis was performed on 667 samples at the Department of Earth Sciences, University of Oxford. The equipment used was a Rock-Eval 6 Standard Analyzer unit by Vinci Technologies. The analyzer includes a pyrolysis and oxidation oven in which homogenized, powdered bulk rock samples are incrementally heated to 300–650 and 300–850°C for the pyrolysis and oxidation ovens, respectively. The

flame ionization detector measures the quantity of free volatile hydrocarbons that are present in a sample before cracking (S1 peak) and the hydrocarbons and CO_2 produced during the thermal cracking of insoluble organic matter (kerogen, S2 and S3 peak). After pyrolysis, the residual organic carbon is heated under air in the oxidation oven to determine residual organic carbon and mineral carbon content (S4 and S5 peak), also using the flame ionization detector. The total organic carbon (TOC, in wt%) is calculated as the sum of pyrolyzed organic C and residual organic C. The Hydrogen Index (HI, in mg HC/g TOC) represents the pyrolyzable organics relative to TOC. Oxygen Index (OI, in mg CO_2/g TOC) represents the amount of oxygen relative to TOC. T_{max} (°C) is the temperature with the highest rate of hydrocarbon generation during pyrolysis temperature (recorded at S2 peak), and is used as a thermal maturity parameter. The mineral carbon (wt%, TIC - total inorganic carbon) is calculated from the sum of pyrolysed and oxidized mineral carbon and was converted to calcium carbonate ($CaCO_3$). Laboratory procedures after Behar et al. (2) were followed. The in-house standard SAB134 (Blue Lias organic-rich marl) was regularly measured (every 8 to 10 samples). The standard deviation on TOC and HI of the in-house standard (SAB134) was 0.11% and ±29.95 mg HC/g TOC, respectively. The standard deviation for T_{max}, OI and TIC were at 1.9°C, 2.8 mg CO_2/g TOC and 0.25%, respectively. The long-term average TOC of the international reference standard IFP 160000 is 3.27%, with a standard deviation of 0.04% (the TOC content of the IFP 160000 standard is referenced at 3.28±0.14 wt%).

2.2 ORGANIC PETROGRAPHY

Optical petrographic rock analysis was performed at the Department of Earth Sciences, University of Oxford, UK. A total of 14 samples from Hettangian,

Sinemurian and upper Pliensbachian strata were analyzed. The samples were mounted in epoxy resin and polished according to the method described by Gorbanenko (3). The polished blocks were analyzed with a Leica DMRX – MPVSP microscope photometer, using both reflected white light and fluorescence illumination under oil immersion at a magnification of ×500. Maceral analysis was undertaken with the point-count method (Peclon counting system) based on 1000 individual determinations per sample. The nomenclature of Taylor (4) was used for the macerals of the vitrinite and inertinite groups, and that of Hutton (5) for the liptinite group macerals. Results are presented in vol% relative to the total organic carbon content.

2.3 $\delta^{13}C_{TOC}$ ANALYSIS

About 1–2 g of homogenized powdered bulk rock sample were treated with ~40 ml of 3 M hydrochloric acid (HCl) and left in a warm water bath (~60°C) for about 2 hours. Samples were then centrifuged and the HCl decanted. Carbonate-rich samples were treated with 3 M HCl a second time. The samples were then rinsed with deionized water until neutral pH was reached, and dried in an oven overnight at ~40°C. Dried samples were homogenized in an agate pestle and mortar and weighed into tin capsules (~10 mg of sample, aiming for 25µg pure carbon). The $\delta^{13}C_{TOC}$ analysis was performed at the NERC Isotope Geosciences Facilities, British Geological Survey, Keyworth, Nottingham (United Kingdom) by combustion in a Costech Elemental Analyser (EA) online to a VG TripleTrap and Optima dual-inlet mass spectrometer. The $\delta^{13}C_{TOC}$ values were calibrated to the VPDB scale using in-house standards, which have been calibrated against international standards (NBS-18, NBS-19 and NBS-22). Replicate analysis of the in-house standards gave a precision of ± <0.1‰ (1 SD).

2.4 $\delta^{13}C_{wood}$ ANALYSIS

Macroscopic fossil plant material was extracted from reserve bags using a metal preparation needle or scalpel, resulting in 97 samples. Extracted wood and leaf fragments were treated with ~1 ml of dilute nitric acid (2 % vol/vol) for approximately 12 hours to dissolve any carbonate remains from attached rock matrix and calcite and other carbonate minerals that might have impregnated the wood or precipitated in cracks. The samples were washed with deionized water at least 5 times. The residual liquid remaining after the washing steps was evaporated in an oven at 50°C within ~12 hours.

The resulting pure wood fragments were gently crushed where necessary and a target amount of 400 to 600 µg of material weighed (at 1 µg precision) transferred into tin capsules for mass spectrometry. Resulting sample weights were 53 to 830 µg, with amounts of < 400 µg in 18 of 110 analyzed samples dictated by sample availability. All samples were measured with a Sercon Integra Gas Source Isotope Ratio Mass Spectrometer, housed at the University of Exeter Penryn Campus. The $\delta^{13}C_{wood}$ values were calibrated to the VPDB scale using in-house standards (Alanine and Bovine Liver), which have been calibrated against international certified standard materials and accuracy checked by running in-house reference materials from the BGS. Reproducibility of the data as measured by the double standard deviation (2 s.d.) of 16 Bovine Liver and 32 Alanine standards is 0.06 ‰ for $\delta^{13}C_{wood}$. Analyses of Alanine yielded a much wider spread of carbon content, because Alanine was pipetted onto an absorbent material rather than weighed like the other unknowns and standards used for mass spectrometry.

2.5 SPECTRAL AND TIME-SERIES ANALYSIS

Spectral and time-series analysis on the Mochras $\delta^{13}C_{TOC}$ record was aimed to test whether the medium-amplitude carbon isotope excursions (CIEs) were paced by long-eccentricity (405-ky) orbital parameters. Data preparation and spectral and time-series analysis were performed using the R Package for Astrochronology, version 0.3.1 (6) on $\delta^{13}C_{TOC}$ data from the Hettangian to Pliensbachian $\delta^{13}C_{TOC}$ data of this study, combined with data from the upper Pliensbachian and lowermost Toarcian (7) of the core, together covering the stratigraphy between 1890 and 855 mbs. The lower part of the *planorbis* Zone (1906.7 to 1890 mbs) was excluded from the analysis, as it is comparatively thin. Moreover, there is a possible stratigraphic gap and associated hiatus at the sharp lithological change at the inferred Triassic–Jurassic boundary.

Analysis of the full-length data set appears unsuitable for defining the dominant frequencies of the $\delta^{13}C_{TOC}$ shifts in the Mochras record as the frequency range of dominant spectral components is changing to shorter frequencies along the dataset as shown by wavelet analysis (Figure S2). This trend is ascribed to sedimentation rate changes. Furthermore, interpolation of the full data set to equal sample spacing adds a bias to the data, creating a considerable number of artificial data points within the intervals of low data resolution (Hettangian to upper Sinemurian), and removing data in the higher-resolution intervals in the lower and upper Pliensbachian (see Figure S1 for sample resolution).

In order to precisely determine the dominant spectral peaks in the $\delta^{13}C_{TOC}$ record and to avoid sample biases such as aliasing, the $\delta^{13}C_{TOC}$ record has been divided into three individual segments based on the wavelet analysis of the full data set (Figure S2, S3), as well as on differences in the original sample spacing, with the aim being to reduce the number of artificial data points during interpolation to equal

sample spacing. The individual segments overlap slightly to avoid artificial peaks at the end-points during spectral analysis. Each segment has been manipulated to the average sample spacing of the grouped data set using linear interpolation.

Spectral analysis on the individual segments was performed using 3π multi-taper spectral analysis with robust red-noise model. Each segment shows a dominant spectral peaks in a wide range of frequencies, some of these overlap with those identified in elemental Ca concentrations (45.4–8 m, 8) and the digitized gamma-ray log (32 m, 9), of the uppermost Sinemurian to Pliensbachian strata of the Mochras core, which have been interpreted to correspond to long eccentricity (405-ky) cycles.

The average spectral misfit (ASM) method for astrochronologic testing (10, 11) was used to evaluate the dominant spectral peaks for each segment (Null-hypothesis test of orbital influence). For segment 1 and 2 all spectral peaks ≥95% MTM harmonic F-test confidence level were evaluated for ASM, utilizing 10,000 Monte Carlo simulations. For segment 3 spectral peaks ≥90% MTM harmonic F-test confidence level were evaluated. Predicted orbital periods (E1=404.8 ky, e1=132.4 ky, e2=99.8 ky, o1=39.9 kry, p1=24.1 ky) were determined from (Ref. 12). Precession (p1) was not used in ASM testing of segment 1 due to lower sample spacing. Average sedimentation rate for the studied interval is estimated to ~5.7 cm/ky, based on the total thickness of the studied section and the duration of the studied interval based on radioisotoic ages (13-18). ASM results were tested for sediment accumulation rates between 2 and 10 cm/ky for segment 1 and 2, and 1 and 6 cm/ky for segment 3, which corresponds to a stratigraphic interval in which a lower sedimentation rate is expected (8). Results with Null Hypothesis (H_0) significance level of ≤0.5% were identified (Figure S3).

The ratios of statistically significant peaks identified as E1, e1, o1, and p1 (see Figure S3) are similar to the ratio 20:5:2(:1) associated with orbital long eccentricity, short

eccentricity, obliquity (and precession) target frequencies (Segment 1: 18:4.6:1.8, Segment 2: 17.3:4:1.6:1.2, segment 3: 18:5.2:2.2:0.6). Furthermore, the dominant spectral peaks identified as 405-ky cycles in the upper Sinemurian through Pliensbachian are in comparable range to the spectral components found in elemental calcium concentrations and gamma ray logs in previous studies (8, 9).

In order to generate a relative (floating) timescale, individual 405-ky orbital cycles were allocated to the $\delta^{13}C_{TOC}$ record based on the dominant spectral components (Figure S2). The data within each defined cycle was interpolated to represent 405-ky using linear interpolation.

Uncertainties occur at the stratigraphic interval corresponding to the *oxynotum* Zone (~1380 to 1280 mbs) where the spectral signal is relatively weak. Several coring and associated sampling gaps, transition between samples referring to a depth-interval to core-slab samples, and the presence of a fault potentially cutting out some strata may inhibit the determination of a strong spectral signal in this interval.

Dominant spectral peaks in the tuned data series were identified by 3π multi-taper spectral analysis with robust red-noise model (Figure 4 of the main text). The dominant spectral peaks correspond to amplitude modulations and short eccentricity and obliquity. The data set has been band-pass filtered to extract the 405-ky cycles and compared with the tuned data set after frequencies >3 My have been removed (Figure 4 of the main text). The duration estimates of the Sinemurian stage and Sinemurian ammonite zones were determined on the floating time scale. For the generation of an absolute time scale, the Triassic–Jurassic boundary was defined as 201.4 Ma (13) as anchor point for the Laskar astronomical solution (19) and the tuned biogenic silica data set (20), and the tuned Mochras record and 405-ky filter anchored to the Pliensbachian-Toarcian boundary at 183.7 Ma (14).

3. RESULTS AND DISCUSSION:

3.1 ROCK-EVAL PYROLYSIS – ANALYTICAL UNCERTAINTIES AND MINERAL MATRIX EFFECT

TOC, HI, and OI values obtained by Rock-Eval analysis from organic-lean samples (<0.5 wt% TOC) are considered to be unreliable due to possible analytical uncertainties (no clear S2 and S3 peaks are generated). Hence, TOC, HI and OI values corresponding to samples with TOC <0.5 wt% (indicated as red squares in Figure S4) were excluded from interpretation.

A common factor artificially reducing TOC and HI values during analysis is the matrix retention of hydrocarbons in the presence of clay minerals (mineral matrix effect) (21), and CO_2 generated from carbonate may lead to elevated OI values (22). The mineral matrix effect is largest in organic-lean samples and those containing abundant clay minerals (especially illite) in the mineral matrix (23). Based on lithological descriptions (22, 24) and observations during sampling for this study, higher amounts of clay minerals are likely to occur in the mudstones present in the lower succession (Hettangian to lower Sinemurian) of the Mochras core where the sediments appear softer and darker compared to the carbonate-rich upper Sinemurian and Pliensbachian strata. This difference suggests a likely greater mineral matrix effect in the stratigraphically lower part of the Mochras core. There, low TOC and HI values (~1.5wt% and ~80 mg HC/g TOC on average, respectively), and the gradual increase in TOC and HI values along the Sinemurian–Pliensbachian transition (2.6wt%, up to 380 mg HC/g TOC, respectively), could therefore be a function of retention within the mineral matrix.

S_2 *versus* TOC plots can be used to identify and quantify the mineral matrix effect and inert carbon, as the negative intersection with the S_2 axis indicates the average magnitude of hydrocarbons retained by the mineral matrix (24). The negative *y*-intercept of the composite Mochras data set (-0.97, Figure S5) indicates that some of the low TOC and HI values, as well as elevated OI values in the Mochras core may be an artifact linked to the mineral matrix. The mineral matrix effect of the Hettangian to upper Sinemurian data set appears less dominant (*y*-intercept at -0.11) compared to the upper Sinemurian to Pliensbachian data (*y*-intercept at -0.88). This result might, however, be biased by the relatively limited amount of samples with TOC>1, hampering assessment of the relationship between S2 and TOC over a range of values.

3.2 THERMAL MATURITY

Thermal maturity can affect TOC and HI values due to the migration of generated hydrocarbons. Both Rock-Eval pyrolysis derived T_{max} values (428 °C on average) and Vitrinite reflectance ($R_{0\ max}$ = 0.38–0.63)(25) indicate moderately low maturity for the Hettangian to Pliensbachian strata. The average T_{max} is furthermore likely to be slightly overestimated due to the matrix-retention effect (5–6 °C for Type II kerogen and 10–12°C for Type III kerogen) (26). The generally lower HIs recorded from Hettangian and Sinemurian strata are therefore not related to higher maturity levels in the deeper part of the core.

3.3 ORGANIC MATTER SOURCE AND PRESERVATION

3.3.1 HI *versus* OI

The van Krevelen-type plot (HI *versus* OI, Figure S5) of the Mochras bulk sediments indicates relatively mature Type III (0–200 mg HC/g TOC, typically sourced from terrestrial organic matter) and Type II (200–400 mg HC/g TOC mixed marine and terrestrial) organic matter (27). Only four samples can be classified as Type I (marine, 400–600 mg HC/g TOC).

Degraded marine organic matter commonly shows a pyrolysis signature almost indistinguishable from terrestrial material (21). Oxygen availability during early diagenesis in the sediment (and likely also in the water column during sinking of the organic particles) can greatly compromise the HI (28), making it difficult to separate source and preservational effect. Samples with TOC > 1 wt% show decreasing OI values with increasing TOC, which may indicate that the organic matter preserved in samples characterized by higher TOC values are less oxidized, with better preserved HI values.

1.1.1 ORGANIC PETROGRAPHY

Microscopic evaluation of the organic matter was used to determine the organic constituents preserved in the Mochras core and to evaluate the degree of oxidation/degradation of the organic matter. A total of 14 samples have been analyzed, giving a broad overview on the organic constituents. The predominant organic component of the analyzed Mochras samples analyzed is liptodetrinite (Figure S6, S7). Liptodetrinite is thought to be produced from the physical breakdown of different marine algae or terrestrially derived liptinites such as sporinite and cutinite derived from the outer wall of spores and pollen, as well as leaves and stems (21) and

is associated with high levels of water-column oxidation (29). The highly degraded shape of liptodetrinite macerals observed in Mochras (Figure S6) suggests fragile marine organic matter as primary source. Furthermore, the fluorescence of terrestrial organic matter should decrease with greater biodegradation (30). The fluorescence of liptodetrinites in the Mochras core instead appears higher compared to intact terrestrial liptinites, indicating primarily marine precursors.

The highly oxidized nature of the suggestively primarily marine organic components is confirmed by the lack of correlation between the relative amounts of marine versus terrestrial organic matter as determined by microscopic evaluation and $\delta^{13}C_{TOC}$ and HI values (Figure S7). Even samples with high relative amounts of liptodetrinite (72–94vol.%, e.g., samples 1747.2, 1656.3 and 1392.9 mbs, Figure S7) comprise HI values in a range generally associated with terrestrially derived organic matter (65–83 mg HC/g TOC). Based on the organic petrography it is thus evident that HI values generated by Rock-Eval pyrolysis are highly compromised and not indicative of the source of organic matter.

Samples corresponding to the upper Pliensbachian strata contain higher relative amounts of bituminite, and some samples include higher amounts of alginite compared to the samples corresponding to Hettangian and Sinemurian strata (Figure S7). Bituminite, also known as amorphous organic matter (AOM), is the product of bacterial decomposition of algae and faunal plankton (31) under low-energy anoxic conditions (29). The presence of bituminite in samples therefore indicates low-oxygen bottom water conditions during the Pliensbachian.

1.2 EFFECTS OF SOURCE OF ORGANIC MATTER, ORGANIC-MATTER DEGRADATION AND PRESERVATION ON $\delta^{13}C_{TOC}$

1.2.1 δ^{13}C *versus* HI

A negative linear relationship between hydrogen indices indicative of the approximate source and source mixture of organic matter and compound-specific isotope values can generally be used to determine the impact of organic-matter changes on shifts in the $\delta^{13}C_{TOC}$ record (21, 32, 33), given that the HI values are well preserved and thus indicative for the primary source. Isotopic variations corresponding to compositional changes of bulk organic matter will plot on, or close to a mixing line defined by the $\delta^{13}C_{TOC}$ and HI values for the marine and terrestrial end-members (21, 32, 33). The HI–$\delta^{13}C_{TOC}$ plot of the composite Mochras dataset indicates two main trends, one of which comprises paired HI–$\delta^{13}C_{TOC}$ data plotting along the suggested background values and might indicate that shifts in $\delta^{13}C_{TOC}$ may be associated with variations in the primary source of the organic matter. The second group, corresponding to paired HI–$\delta^{13}C_{TOC}$ data related to the main large-scale CIE intervals, plots on a steeper slope, indicating that changes in $\delta^{13}C_{TOC}$ not being related or only marginally related to changes in HI, with the exception of the upper Pliensbachian *margaritatus* zone positive excursion (Figure S5). As discussed above, the HI values are likely not indicative for relative changes in the source and source mixture of the bulk organic matter but rather compromised by oxic degradation. A similar plot testing a link between $\delta^{13}C_{TOC}$ and the relative amount of marine/terrestrial components in the bulk organic matter based on microscopic evaluation (Figure S7) does not show a correlation between the relative amount of marine/terrestrial organic constituents and isotope values. Although data are available for the Sinemurian–Pliensbachian transition, which is marked by a large ~4‰ negative CIE, it appears unlikely that an

increase in marine organic matter contributes to the magnitude of shift as marine organic matter comprises the predominant amount of the bulk organic matter in both, the underlying and above strata. It is, however, noteworthy that the available data points (14 samples analyzed) may not be representative enough to resolve the impact of relative changes in the primary organic matter source, or preferential preservation of more refractory terrestrial organic matter (signature characterized by more positive $\delta^{13}C_{TOC}$ values compared with marine organic matter) on the medium-amplitude $\delta^{13}C_{TOC}$ fluctuations.

If changes in the preservation of organic matter had affected the $\delta^{13}C_{TOC}$ signature of the bulk sediment in Mochras, a linear relationship between $\delta^{13}C_{TOC}$ and TOC would be expected, with poorly preserved low-TOC samples showing a $\delta^{13}C_{TOC}$ signature distinctly different from better-preserved (higher TOC) samples. For the TOC–$\delta^{13}C_{TOC}$ cross-plot of the Mochras data, intervals were selected where the $\delta^{13}C_{TOC}$ is not affected by major long-term shifts (1060–950 mbs and 1550–1370 mbs). Within these intervals, the data plot shows no particular correlation between TOC and $\delta^{13}C_{TOC}$ (Figure S5).

The effects of oxic degradation of organic matter in the water column and in the sediment on the bulk organic $\delta^{13}C_{TOC}$ composition are thus far not fully understood. The diagenetic isotope fractionation as a result of prolonged oxic-suboxic degradation in the sediment is assumed to lead to lighter isotope values of ≤2–3 ‰ (21). This magnitude of shift lies in the range of the consecutive appearing medium-amplitude shifts recorded in Mochras, suggesting oxic-suboxic degradation as possible driving mechanism. The magnitude of individual CIEs appears larger in the Hettangian to upper Sinemurian stratigraphic interval, which, based on the organic matter assemblage, is associated with better-oxygenated bottom waters compared to

the upper Sinemurian to Pliensbachian (34, and this study). As similar CIEs have been recorded in $\delta^{13}C_{carb}$ (see discussion in the main text) it appears unlikely that the medium-amplitude CIEs in $\delta^{13}C_{TOC}$ are solely driven by changes in preservation.

3.2. CONCLUSION

The Mochras bulk sediments comprise highly oxidized organic matter of likely predominantly marine origin. HI and OI values associated with low TOC samples are likely compromised by the mineral matrix effect. HI values are generally low due to oxidation in the water column and sediment. The partial overprint of the organic matter did likely not affect the isotopic signature of the bulk sediment as large-scale global isotope trends are recorded in the isotopic signature. Whether the medium-scale $\delta^{13}C_{TOC}$ shifts are a primarily isotopic signal, or a result of differential preservation cannot be resolved with the current dataset. Changes in the relative organic matter assemblage and the associated impact on the bulk organic carbon-isotope signature cannot be reconstructed via HI–$\delta^{13}C_{TOC}$ as to the highly compromised HI values, which are not unambiguously indicative for the primary source of the organic matter. No major changes in the relative organic matter assemblage are evident from the maceral assemblage. Furthermore, the $\delta^{13}C_{TOC}$ signal of the Mochras sediments appears not to be biased by thermal maturation.

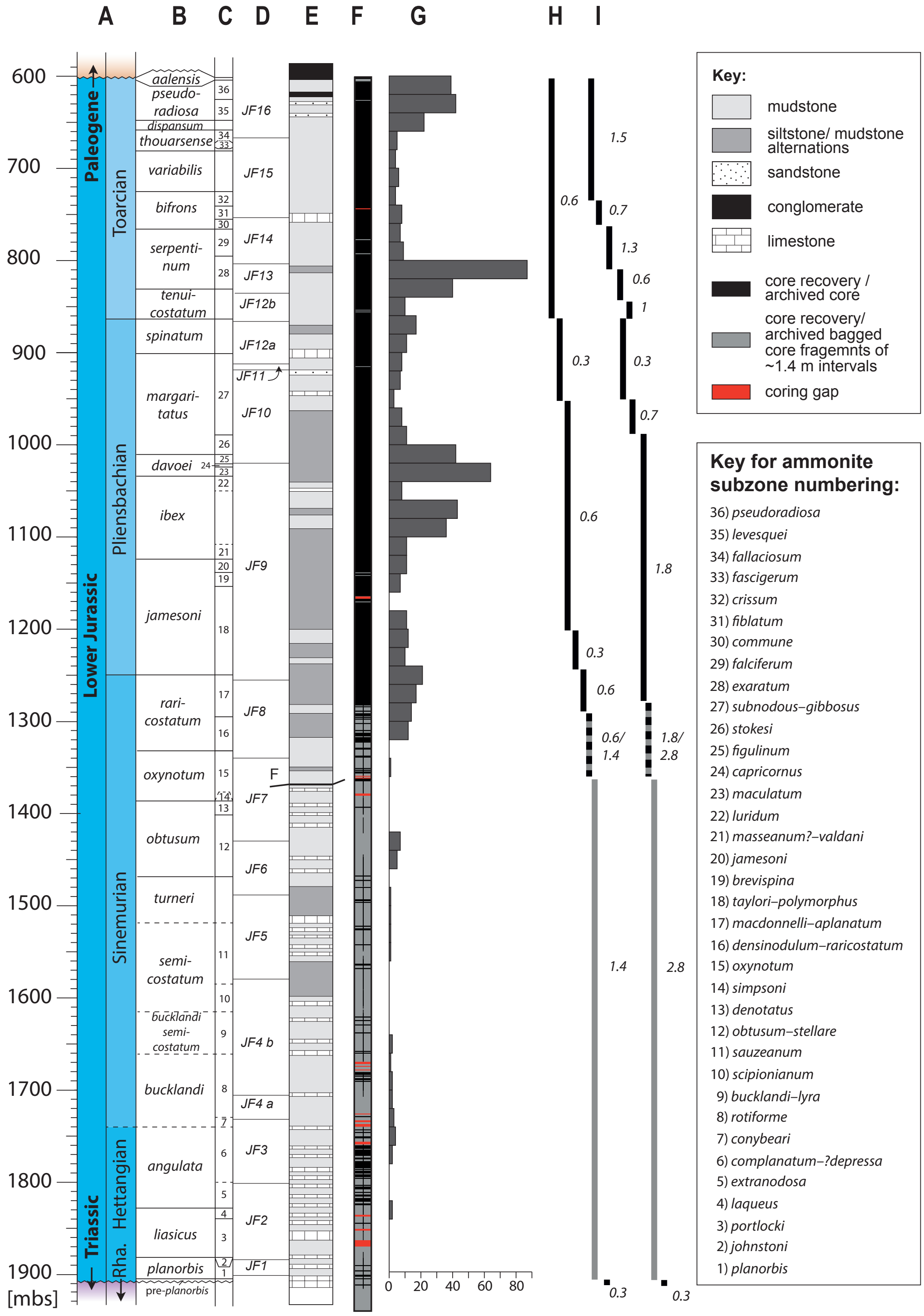

Supplementary Figure S1: Stratigraphy of the Mochras borehole. **A**: Stratigraphy. **B** – **C**: Ammonite zonation and sub-zonation after ref. 35, revised in ref. 36. Key for subzone numbering given in figure. **D**: Foraminiferal biostratigraphy (36). **E**: Litholog (9), key given in figure, F – fault. **F**: core recovery (total recovery 97.6 %); black: archived preserved core (59.1 %), gray: core preserved as aggregated bagged core fragments (reserve collection samples, aggregating ~1.4 m intervals), red: coring gaps and corresponding sampling gaps. **G**: Abundance of registered macroscopic fossil wood specimen. **H**: Average sample spacing of sample sets analyzed for $\delta^{13}C_{TOC}$ **I**: Average sample spacing of sample sets analyzed for Rock-Eval pyrolysis. Figure modified after ref. 9.

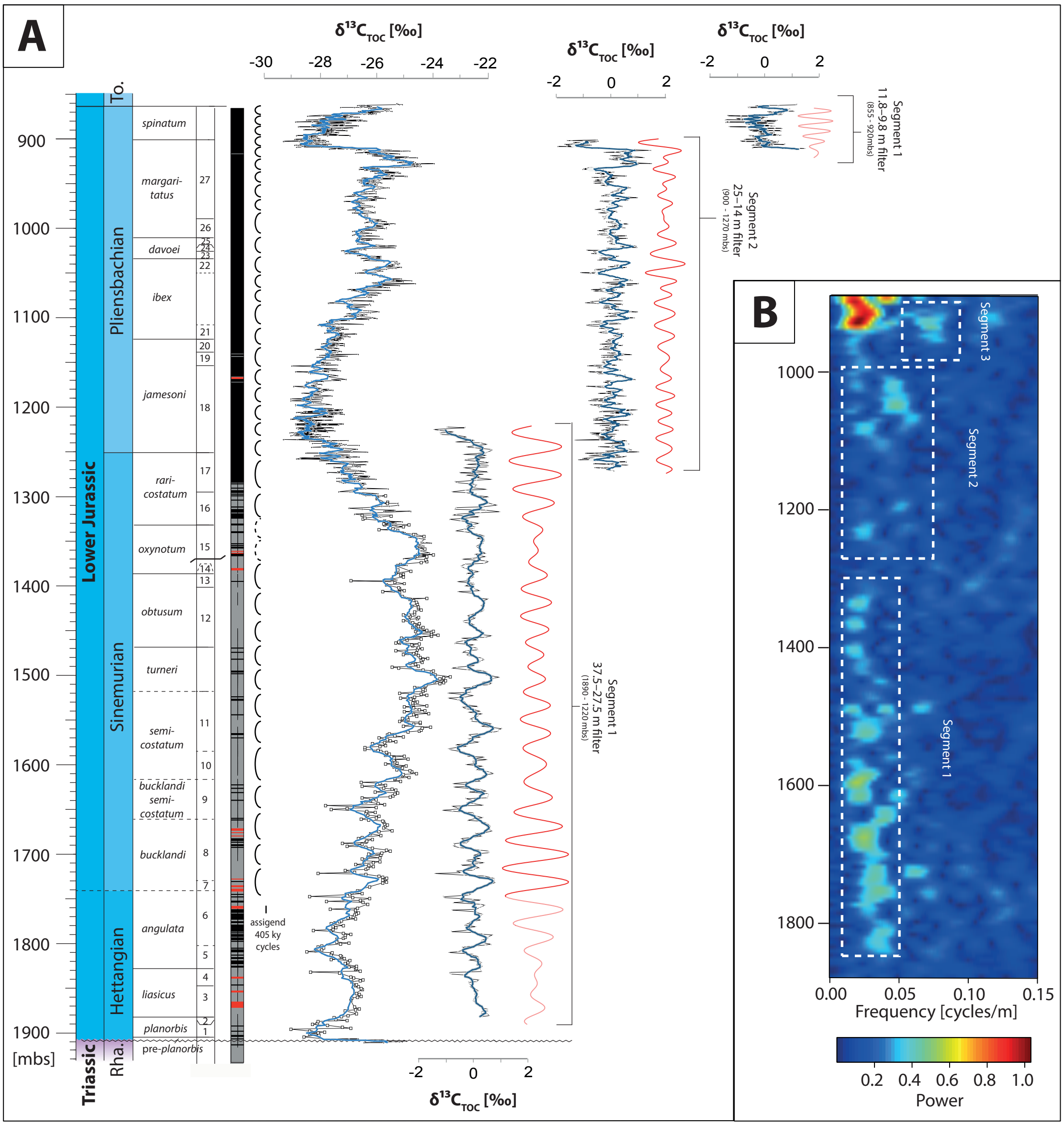

Supplementary Figure S2: Spectral and wavelet analysis of the Mochras $\delta^{13}C_{TOC}$ record of the Hettangian to lowermost Toarcian **A**) $\delta^{13}C_{TOC}$ record, interpolated, high band-pass filtered dataset (frequencies corresponding to 80 m cycles removed from segment 1, and 50 m from segment 2 and 3) and band-pass filter of dominant spectral peaks identified as 405-ky signal for each segment (red, band-pass parameter given in figure). Key to ammonite subzone numbering given in Figure S1. **B**) Wavelet analysis of the Mochras $\delta^{13}C_{TOC}$ record after long frequencies (>100 m) were removed. Note the gradual shift of dominant spectral components towards shorter frequencies up-sequence. Approximate position of segments A to C marked in figure.

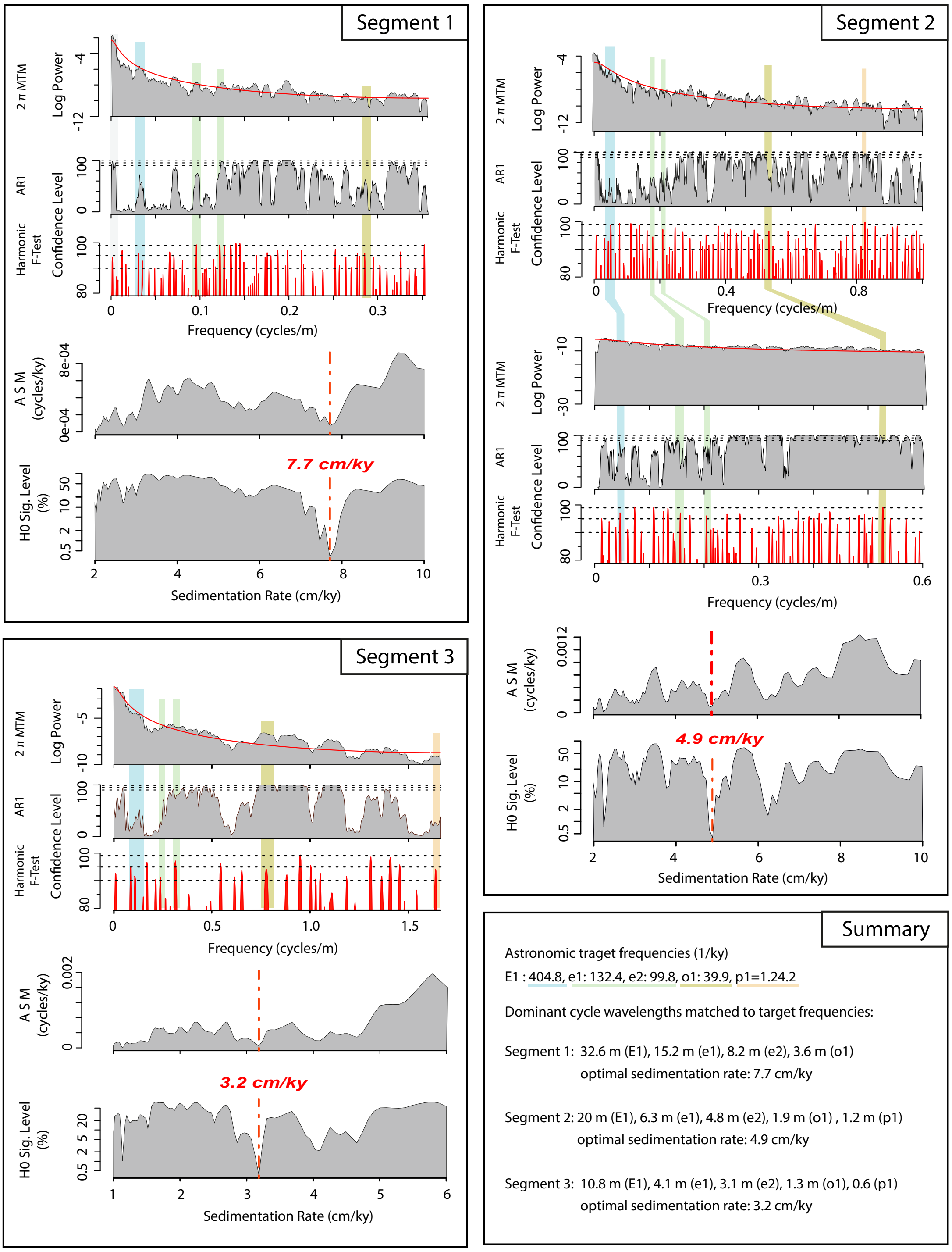

Supplementary Figure S3: Multitaper method (MTM) power spectrum and average spectral misfit test for each of the three data segments. MTM of segment 2 is shown for the full frequency range (upper plot) and for the data set after long and short periodicities (>80, <1.6 m) were removed (lower plot).

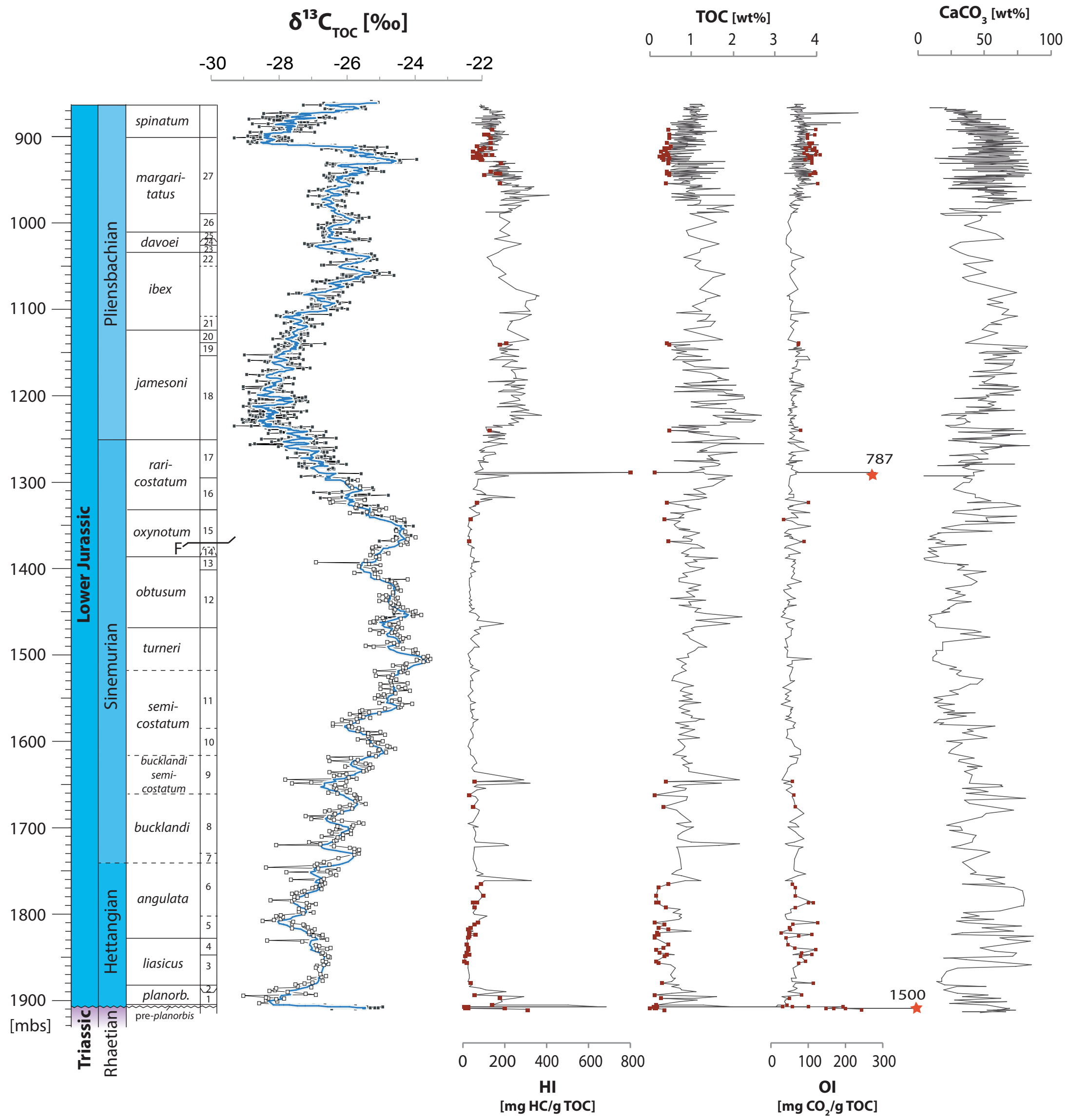

Supplementary Figure S4: $\delta^{13}C_{TOC}$, Hydrogen Index (HI), total organic-matter content (TOC), Oxygen Index (OI) and %calcite ($CaCO_3$, calculated from total inorganic carbon) of the uppermost Rhaetian to Pliensbachian strata. OI outliers are marked with red star, outlier values are given in figure. Samples with TOC values < 0.5 wt% and corresponding HI and OI are marked in red. $\delta^{13}C_{TOC}$ samples shown as white squares indicate samples taken from reserve collection bags, depth refers to mid-point of reserve bag sampling interval. For ammonite subzone nomenclature, see Figure S1.

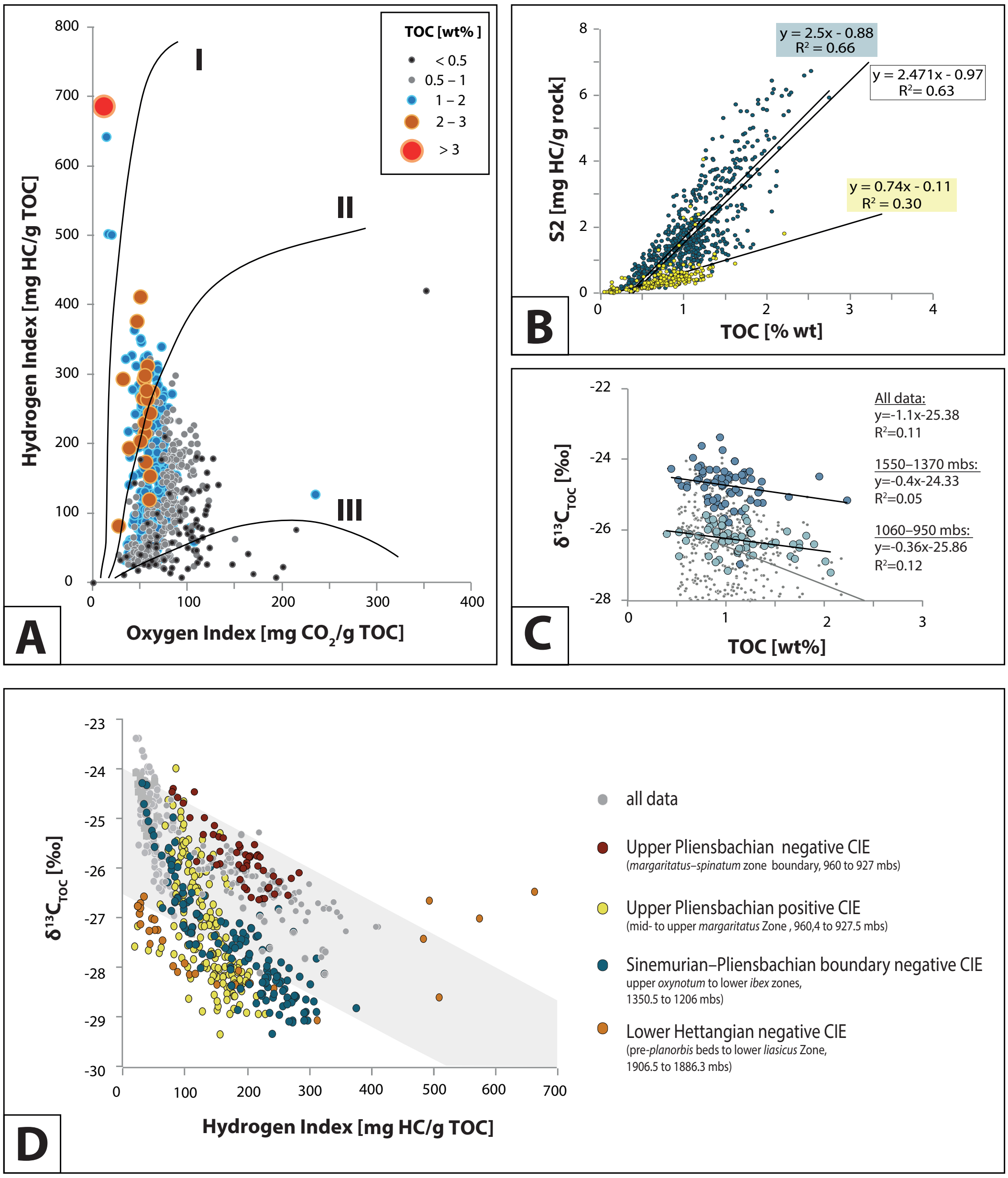

Supplementary Figure S5: Cross-plots of geochemical parameter **A)** van Krevelen-type diagram. Color code and size of circles refer to TOC values **B)** TOC–S2 cross-plot to indicate the matrix retention effect. Yellow circles: data from Hettangian to upper Sinemurian strata (pre-*planorbis* beds to upper *oxynotum* Zone; 1906.7 to 1350 mbs), regression line marked in yellow. Blue circles: Upper Sinemurian to Pliensbachian data (upper *oxynotum* to *spinatum* zones), regression line marked in blue, and regression line for the entire data set (white). **C)** TOC–$\delta^{13}C_{TOC}$ plot. Samples with TOC <0.5 wt% are not included in the plot. Light gray circles represent all data with TOC <0.5 wt%. Blue (1550–1370 mbs) and light blue (1060–950 mbs) circles represent intervals where $\delta^{13}C_{TOC}$ is not affected by major long-term shifts. No particular correlation between TOC and $\delta^{13}C_{TOC}$ is apparent. **D)** HI–$\delta^{13}C_{TOC}$ plot.

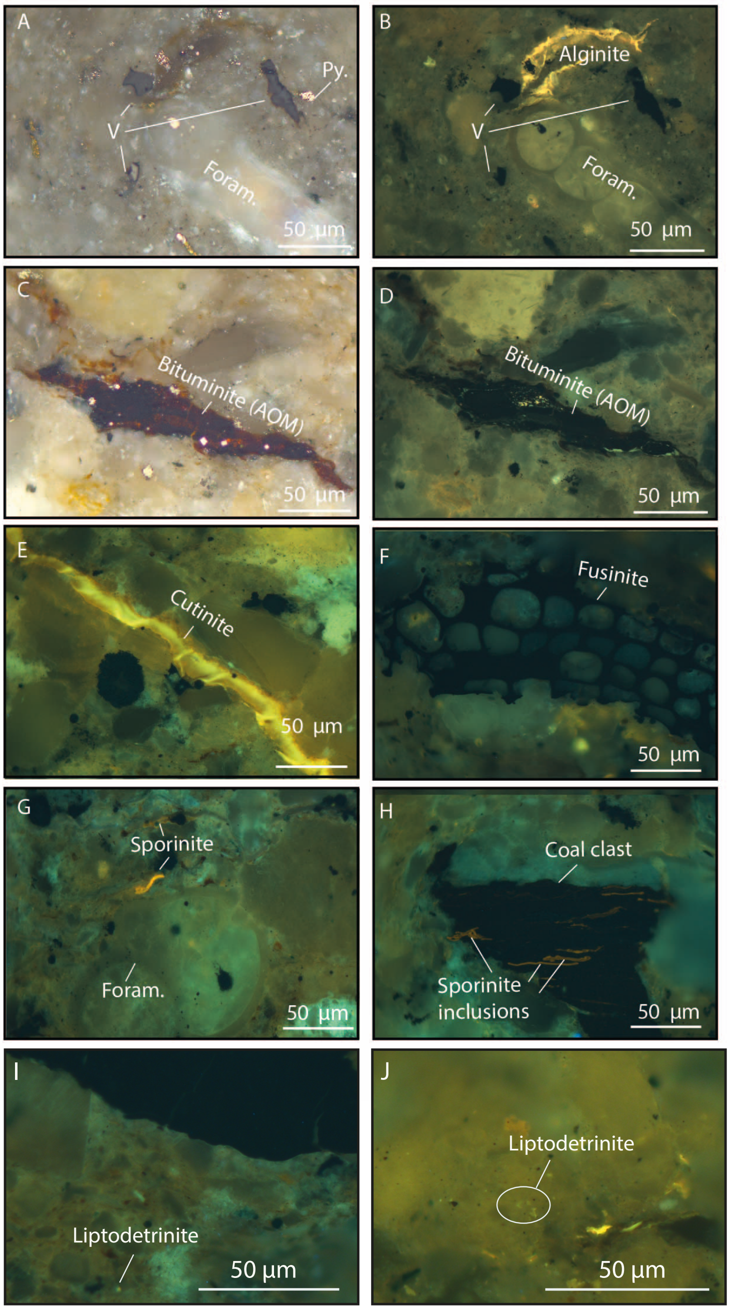

Supplementary Figure S6: Photomicrographs illustrating the organic facies of selected petrographically analyzed samples. **A:** reflected white light photomicrographs of sample 1748.6 mbs, showing vitrinite macerals (V) and a zooclast (foraminifera) **B:** same view in fluorescence mode, oil emersion, showing degraded alginate within the foraminiferal test. Typical microfacies of Hettangian and Sinemurian samples from Mochras. **C:** bituminite (amorphous organic matter, AOM) in reflected white light, sample 973.9 mbs. **D:** same view in fluorescence mode, oil emersion **E:** cutinite in fluorescence mode, oil emersion (sample 901.9 mbs) **F:** well-preserved intertinite (fusinite) fluorescence mode, oil emersion (sample 962.2 mbs). **G:** sporinite in typical microfacies for the upper Pliensbachian strata, fluorescence mode, oil emersion. **H:** coal fragment with sporinite inclusions, typical for the Pliensbachian organic facies. **I:** liptodetrinite (sample 884.7 mbs). **J:** liptodetrinite (sample 941.9 mbs).

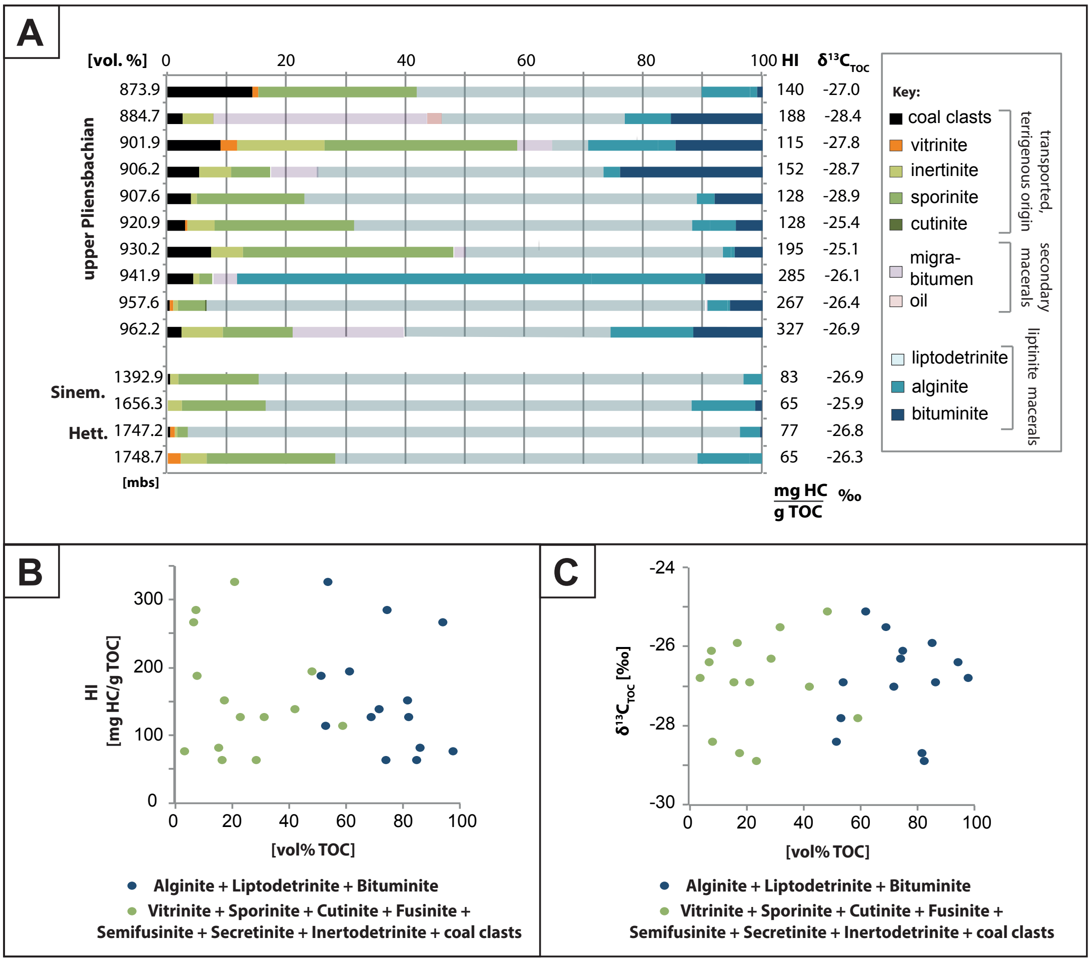

Supplementary Figure S7: Maceral composition and HI of the samples studied from the Hettangian and Sinemurian, and upper Pliensbachian strata of the Mochras core. **A)** Relative abundance of macerals, normalized to total organic matter (vol.%). **B)** Cross-plot between HI and marine organic matter and **B)** Cross-plot between HI and terrestrial organic matter in vol% of TOC. Cross-plots show no correlation between the source of the organic matter and HI.

4. REFERENCES

1. A. W. Woodland, *The Llanbedr (Mochras Farm) Borehole*. A. W. Woodland, Ed., The Llandbedr (Mochras Farm) Borehole (Institute of Geological Sciences, Report No. 71/18, 1971).
2. F. Behar, V. Beaumont, H. D. B. Penteado, Rock-Eval 6 technology: performances and developments. *Oil & Gas Science and Technology* **56**, 111–134 (2001).
3. O. Gorbanenko, A dry polishing technique for the petrographic examination of mudrocks. *International Journal of Coal Geology* **180**, 122–126 (2017). https://doi.org/10.1016/j.coal.2017.03.013.
4. G. H. Taylor, *Organic petrology : a new handbook incorporating some revised parts of Stach's Textbook of coal petrology*. ed D. C. Glick (Gebrüder Borntraeger, Berlin, 1998).
5. A. C. Hutton, Petrographic classification of oil shales. *International Journal of Coal Geology* **8**, 203–231 (1987).
6. S. R. Meyers, Astrochron: an R package for astrochronology (version 0.3.1) (2014).
7. W. Xu *et al.*, Evolution of the Toarcian (Early Jurassic) carbon-cycle and global climatic controls on local sedimentary processes (Cardigan Bay Basin, UK). *Earth and Planetary Science Letters* **484**, 396–411 (2018). https://doi.org/10.1016/j.epsl.2017.12.037.
8. M. Ruhl *et al.*, Astronomical constraints on the duration of the Early Jurassic Pliensbachian Stage and global climatic fluctuations. *Earth and Planetary Science Letters* **455**, 149–165 (2016). https://doi.org/10.1016/j.epsl.2016.08.038.
9. S. P. Hesselbo *et al.*, Mochras borehole revisited: a new global standard for Early Jurassic earth history. *Scientific Drilling* **16**, 81–91 (2013). https://doi.org/10.5194/sd-16-81-2013.
10. S. R. Meyers, B. B. Sageman, M. A. Arthur, Obliquity forcing of organic matter accumulation during Oceanic Anoxic Event 2. *Paleoceanography* **27** (2012). https://doi.org/10.1029/2012PA002286.
11. S. R. Meyers, B. B. Sageman, Quantification of deep-time orbital forcing by average spectral misfit. *American Journal of Science* **307**, 773-792 (2007). https://doi.org/10.2475/05.2007.01.
12. J. Laskar *et al.*, A Long-term Numerical Solution for the Insolation Quantities of the Earth. *Astronomy and Astrophysics* **428** (2004). https://doi.org/10.1051/0004-6361:20041335.
13. J. F. Wotzlaw *et al.*, Towards accurate numerical calibration of the Late Triassic: High-precision U-Pb geochronology constraints on the duration of the Rhaetian. *Geology* **42**, 571–574 (2014). https://doi.org/10.1130/G35612.1.
14. J. G. Ogg, G. M. Ogg, F. M. Gradstein, "Chapter 12 - Jurassic" in *A Concise Geologic Time Scale,* J. G. Ogg, G. M. Ogg, F. M. Gradstein, Eds. (Elsevier, 2016), pp. 167-186. https://doi.org/10.1016/B978-0-444-59467-9.00012-1.
15. L. F. De Lena *et al.*, The driving mechanisms of the carbon cycle perturbations in the late Pliensbachian (Early Jurassic). *Scientific Reports* **9**, 18430 (2019). https://doi.org/10.1038/s41598-019-54593-1.
16. T. R. Them *et al.*, Evidence for rapid weathering response to climatic warming during the Toarcian Oceanic Anoxic Event. *Scientific Reports* **7**, 5003 (2017). https://doi.org/10.1038/s41598-017-05307-y.

17. B. Sell *et al.*, Evaluating the temporal link between the Karoo LIP and climatic–biologic events of the Toarcian Stage with high-precision U–Pb geochronology. *Earth and Planetary Science Letters* **408**, 48–56 (2014). https://doi.org/10.1016/j.epsl.2014.10.008.
18. B. Schoene, J. Guex, A. Bartolini, U. Schaltegger, T. J. Blackburn, Correlating the end-Triassic mass extinction and flood basalt volcanism at the 100 ka level. *Geology* **38**, 387–390 (2010). https://doi.org/10.1130/G30683.1.
19. J. Laskar, A. Fienga, M. Gastineau, H. Manche, La2010: a new orbital solution for the long-term motion of the Earth. *Astronomy & Astrophysics* **532**, A89 (2011). https://doi.org/10.1051/0004-6361/201116836.
20. M. Ikeda, R. Tada, K. Ozaki, Astronomical pacing of the global silica cycle recorded in Mesozoic bedded cherts. *Nature Communications* **8**, 15532 (2017). https://doi.org/10.1038/ncomms15532.
21. R. V. Tyson, Sedimentary Organic Matter. (Charman & Hall, London, 1995), p 615.
22. J. Espitalie, M. Madec, B. Tissot, Role of mineral matrix in kerogen pyrolysis: influence on petroleum generation and migration. *AAPG Bulletin* **64**, 59–66 (1980).
23. J. Espitalie, G. Deroo, F. Marquis, La pyrolyse Rock-Eval et ses applications. Troisième partie. *Revue de l'Institut français du Pétrole* **41**, 73–89 (1986).
24. B. Dahl *et al.*, A new approach to interpreting Rock-Eval S 2 and TOC data for kerogen quality assessment. *Organic Geochemistry* **35**, 1461–1477 (2004). https://doi.org/10.1016/j.orggeochem.2004.07.003.
25. S. P. Holford, P. F. Green, J. P. Turner, Palaeothermal and compaction studies in the Mochras borehole (NW Wales) reveal early Cretaceous and Neogene exhumation and argue against regional Palaeogene uplift in the southern Irish Sea. *Journal of the Geological Society* **162**, 829–840 (2005). https://doi.org/10.1144/0016-764904-118.
26. R. A. Schegg, "Thermal maturity and history of sediments in the North Alpine Foreland Basin", University of Geneva, Switzerland. (1993).
27. B. Tissot, D. Welte, *Petroleum occurrence and formation*. (Springer-Verlag: Heidelberg, 1978).
28. L. M. Pratt, Influence of paleoenvironmental factors on preservation of organic matter in Middle Cretaceous Greenhorn Formation, Pueblo, Colorado. *AAPG Bulletin* **68**, 1146–1159 (1984).
29. O. Gorbanenko, B. Ligouis, Variations of organo-mineral microfacies of Posidonia Shale from the Lower Saxony Basin and the West Netherlands Basin: Application to paleoenvironmental reconstruction. *International Journal of Coal Geology* **152**, 78–99 (2015). https://doi.org/10.1016/j.coal.2015.09.011.
30. U. Langrock, R. Stein, Origin of marine petroleum source rocks from the Late Jurassic to Early Cretaceous Norwegian Greenland Seaway—evidence for stagnation and upwelling. *Marine and Petroleum Geology* **21**, 157–176 (2004). https://doi.org/10.1016/j.marpetgeo.2003.11.011.
31. M. Teichmüller, The genesis of coal from the viewpoint of coal petrology. *International Journal of Coal Geology* **12**, 1–87 (1989). https://doi.org/10.1016/0166-5162(90)90016-R.
32. G. Suan, B. Schootbrugge, T. Adatte, J. Fiebig, W. Oschmann, Calibrating the magnitude of the Toarcian carbon cycle perturbation. *Paleoceanography* **30**, 495-509 (2015).

33. A. Sluijs, G. R. Dickens, Assessing offsets between the $\delta^{13}C$ of sedimentary components and the global exogenic carbon pool across early Paleogene carbon cycle perturbations. *Global Biogeochemical Cycles* **26** (2012). https://doi.org/10.1029/2011GB004224.
34. B. van de Schootbrugge *et al.*, Early Jurassic climate change and the radiation of organic-walled phytoplankton in the Tethys Ocean. *Paleobiology* **31**, 73–97 (2005). https://doi.org/10.1666/0094-8373(2005)031%3C0073:EJCCAT%3E2.0.
35. H. C. Ivimey-Cook, "Stratigraphical palaeontology of the Lower Jurassic of the Llanbedr (Mochras Farm) Borehole" in *The Llandbedr (Mochras Farm) Borehole,* A. W. Woodland, Ed. (Institute of Geological Sciences, Report No. 71/18, 1971), pp. 87–92.
36. P. Copestake, B. Johnson, Lower Jurassic Foraminifera from the Llanbedr (Mochras Farm) Borehole, North Wales, UK. *Monograph of the Palaeontographical Society* **167**, 1–403 (2014). https://doi.org/10.5061/dryad.75j12.
